# A cholinergic neuroskeletal interface promotes bone formation during postnatal growth and exercise

**DOI:** 10.1016/j.stem.2022.02.008

**Published:** 2022-04-07

**Authors:** Stephen Gadomski, Claire Fielding, Andrés García-García, Claudia Korn, Chrysa Kapeni, Sadaf Ashraf, Javier Villadiego, Raquel del Toro, Olivia Domingues, Jeremy N. Skepper, Tatiana Michel, Jacques Zimmer, Regine Sendtner, Scott Dillon, Kenneth E.S. Poole, Gill Holdsworth, Michael Sendtner, Juan J. Toledo-Aral, Cosimo De Bari, Andrew W. McCaskie, Pamela G. Robey, Simón Méndez-Ferrer

**Affiliations:** 1 Wellcome-MRC Cambridge Stem Cell Institute, Cambridge CB2 0AW, UK; 2 Department of Hematology, University of Cambridge, Cambridge CB2 0AW, UK; 3 National Health Service Blood and Transplant, Cambridge Biomedical Campus, Cambridge CB2 0AW, UK; 4 Skeletal Biology Section, National Institute of Dental and Craniofacial Research, National Institutes of Health, Department of Health and Human Services, Bethesda, MD 20892, USA; 5 NIH Oxford-Cambridge Scholars Program in Partnership with Medical University of South Carolina, Charleston, SC 29425, USA; 6 Arthritis and Regenerative Medicine Laboratory, Aberdeen Centre for Arthritis and Musculoskeletal Health, Institute of Medical Sciences, University of Aberdeen, Aberdeen AB25 2ZD, UK; 7 Instituto de Biomedicina de Sevilla-IBiS (Hospitales Universitarios Virgen del Rocío y Macarena/CSIC/Universidad de Sevilla), 41013 Seville, Spain; 8 Departamento de Fisiología Médica y Biofísica, Universidad de Sevilla, 41009 Seville, Spain; 9 Centro de Investigación Biomédica en Red sobre Enfermedades Neurodegenerativas, (CIBERNED), Madrid 28029, Spain; 10 Department of Infection and Immunity, Luxembourg Institute of Health, 4354 Esch-sur Alzette, Luxembourg; 11 Department of Physiology, Development, and Neuroscience, Cambridge Advanced Imaging Centre, University of Cambridge, Cambridge CB2 3DY, UK; 12 Institute of Clinical Neurobiology, University Hospital of Wuerzburg, 97080 Wuerzburg, Germany; 13 Cambridge NIHR Biomedical Research Centre, Department of Medicine, University of Cambridge, Cambridge CB2 0QQ, UK; 14 UCB Pharma, 208 Bath Road, Slough SL1 3WE, UK; 15 Department of Surgery, School of Clinical Medicine, University of Cambridge, Cambridge CB2 0QQ, UK

**Keywords:** cholinergic, sympathetic, osteocyte, autonomic, development, skeletal, bone, exercise, neuroskeletal, anabolic

## Abstract

The autonomic nervous system is a master regulator of homeostatic processes and stress responses. Sympathetic noradrenergic nerve fibers decrease bone mass, but the role of cholinergic signaling in bone has remained largely unknown. Here, we describe that early postnatally, a subset of sympathetic nerve fibers undergoes an interleukin-6 (IL-6)-induced cholinergic switch upon contacting the bone. A neurotrophic dependency mediated through GDNF-family receptor-α2 (GFRα2) and its ligand, neurturin (NRTN), is established between sympathetic cholinergic fibers and bone-embedded osteocytes, which require cholinergic innervation for their survival and connectivity. Bone-lining osteoprogenitors amplify and propagate cholinergic signals in the bone marrow (BM). Moderate exercise augments trabecular bone partly through an IL-6-dependent expansion of sympathetic cholinergic nerve fibers. Consequently, loss of cholinergic skeletal innervation reduces osteocyte survival and function, causing osteopenia and impaired skeletal adaptation to moderate exercise. These results uncover a cholinergic neuro-osteocyte interface that regulates skeletogenesis and skeletal turnover through bone-anabolic effects.

## Highlights

• IL-6 induces a cholinergic switch of sympathetic neurons contacting bone postnatally
• Neurturin-GFRα2 pathway maintains cholinergic neuro-osteocyte coupling and survival
• Bone-lining osteoprogenitors amplify cholinergic signaling in bone and bone marrow
• IL-6-induced cholinergic signaling has a bone-anabolic effect during moderate exercise

## Graphical abstract

**Figure:**
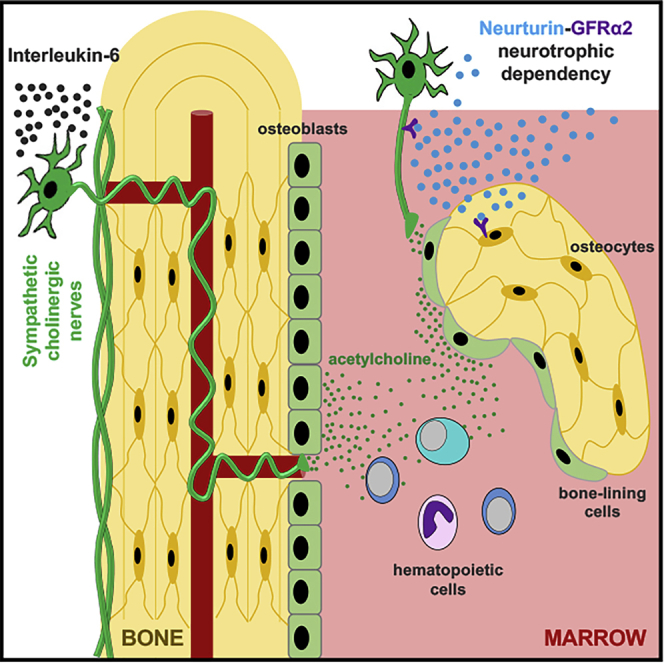

## Teaser

Gadomski et al. describe a neuro-osteocyte interface whereby sympathetic cholinergic neurons support bone-embedded osteocytes through the GFRα2 neurotrophic pathway. Developmentally, these sympathetic neurons undergo a neurotransmitter switch from adrenergic to cholinergic—a process that is induced by interleukin-6 and is dynamically enhanced by physical activity to increase bone mass.

## Introduction

The two branches of the autonomic nervous system, sympathetic and parasympathetic, normally use the postsynaptic neurotransmitters norepinephrine (noradrenergic) and acetylcholine (ACh) (cholinergic), respectively. However, some embryonic sympathetic neurons exhibit cholinergic features, but their frequency gradually diminishes to ∼4% of sympathetic neurons by birth (Huang et al., 2013; Schäfer et al., 1997). It is unclear whether these early target-independent sympathetic cholinergic neurons overlap with sympathetic neurons that become cholinergic postnatally (Schütz et al., 2015). This “cholinergic switch” (Wolinsky and Patterson, 1983) of sympathetic neurons occurs during the first postnatal weeks in rodents (Guidry and Landis, 1998) and was characterized *in vivo* in the sweat glands and the periosteum (Asmus et al., 2000; Hohmann et al., 1986). In bone, the cholinergic switch resembles the neurotransmitter change in sweat glands (Habecker and Landis, 1994), since it requires initially noradrenergic activity, ensuing secretion of yet unidentified cholinergic differentiation factors, ACh release, and maturation of both the target organ and its cholinergic innervation. Further, the role of skeletal cholinergic fibers in bone development and remodeling remains largely unexplored. One study suggested that cholinergic fibers innervate bone and transmit anabolic signals from the brain (Bajayo et al., 2012). Alternatively, parasympathetic signals can promote bone formation by antagonizing bone-catabolic sympathetic noradrenergic signals in the brain via muscarinic ACh receptors (Shi et al., 2010). Therefore, we sought to identify the factor driving the cholinergic switch *in vivo* and examine the functional significance of skeletal sympathetic cholinergic fibers.

Here, we have identified IL-6 as a driver of the cholinergic switch of bone-associated sympathetic neurons during postnatal development and a promoter of cholinergic signaling in response to physical activity during adolescence. A neurotrophic dependency is established between cholinergic nerve fibers and osteocytes relying on the GFRα2-neurturin (NRTN) axis. Bone-lining osteoprogenitors connected with the osteocyte network transmit and amplify the cholinergic signals in the bone marrow (BM). Lack of skeletal cholinergic nerve fibers causes osteocyte atrophy and osteopenia due to reduced bone formation, while increased IL-6 during exercise drives expansion of bone-anabolic cholinergic fibers. These results uncover a dynamic bone-anabolic function of sympathetic cholinergic fibers coupled with the osteocyte network.

## Results

### Cholinergic nerve fibers and bone-lining cells in bone and BM

**Figure 1:**
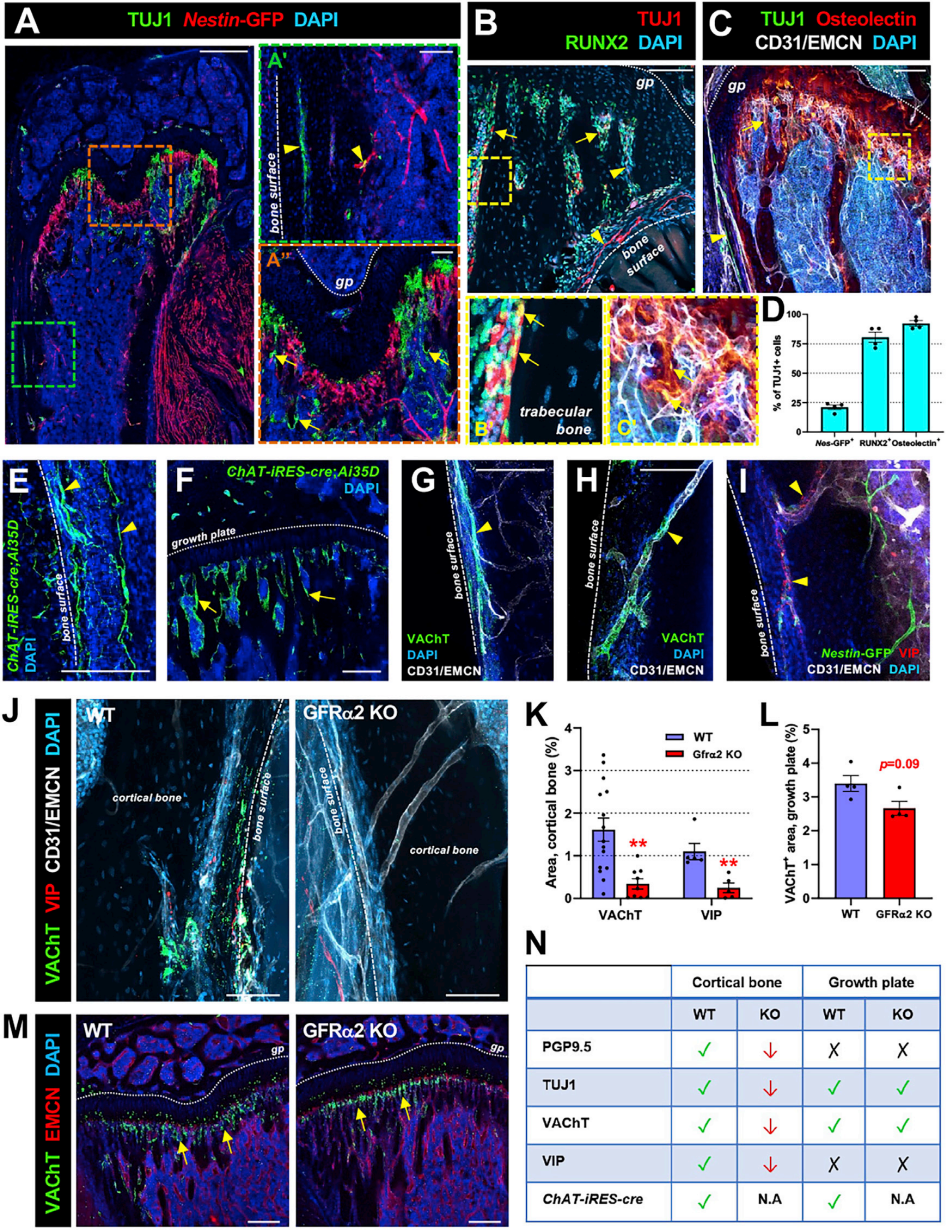
Characterization of the cholinergic system in bone (A) Immunofluorescence of pan-neural TUJ1 in *Nes-GFP* femur. Insets show bone (A′) and growth plate (Aʺ) areas. Scale bars, 500 μm (A) and 100 μm (Aʹ and Aʺ). (B and C) Immunofluorescence of osteolineage markers in WT long bones with high-magnification insets. (D) Frequency of TUJ1^+^ cells among *Nestin*-GFP^+^ and osteolineage cells expressing RUNX2 or osteolectin. (E and F) Genetic tracing of cholinergic cells in (E) cortical bone and (F) growth plate of *ChAT-IRES-cre;Ai35D* mice. Scale bars, 200 μm. See also Figure S1C. (G and H) Immunofluorescence of VAChT^+^ cholinergic nerve fibers in (G) periosteum and (H) cortical bone in WT femur. See also Figures S1E–S1G. (I and J) Immunofluorescence of VIP^+^ or VAChT^+^ cholinergic nerve fibers in cortical bone of (I) a *Nes-GFP* mouse, or (J) WT or GFRα2 KO mice. (K and L) Area covered by (K) VAChT^+^ or VIP^+^ cholinergic nerve fibers in cortical bone or (L) VAChT^+^ cells near the growth plate of WT or GFRα2 KO mice. (M) Immunofluorescence of VAChT^+^ cells in the growth plate of WT or GFRα2 KO tibias. Scale bars, 200 μm. (N) Summary of changes in pan-neural and cholinergic markers in GFRα2 KO mice. N.A., not assessed. (B, C, and G–J) Scale bars, 100 μm. (D, K, and L) Data are mean ± SEM; ^∗∗^p < 0.01, unpaired two-tailed t test. (A–I) Arrowheads depict nerve fiber staining and arrows depict non-neural staining. (A–J and M) Nuclei were counterstained with DAPI (blue). EMCN, endomucin.

We performed immunofluorescence studies to map nerve fibers in bone and BM. 3D imaging of wild-type (WT) and *Nes-gfp* transgenic mice—in which a subset of GFP-labeled cells marks skeletal stem cells (SSCs) (Méndez-Ferrer et al., 2010)—showed protein gene product 9.5 (PGP9.5)^+^ nerve fibers in cortical bone, near the growth plate, and throughout the skull (Figures S1A and S1B). Unexpectedly, the pan-neural marker β-III tubulin (TUJ1) did not only label nerve fibers (Figures 1A–1C, arrowheads) but also osteolineage cells expressing runt-related transcription factor 2 (RUNX2) or osteolectin (Yue et al., 2016) (Figures 1A–1D, arrows). *Choline acteyltransferase (ChAT)-IRES-cre* mice (Rossi et al., 2011) were intercrossed with *Ai35D* reporter mice (Madisen et al., 2012) to genetically label cholinergic neurons. Resembling TUJ1, *ChAT-IRES-Cre* tracing did not only mark nerve fibers in cranial (Figure S1C) and femoral (Figures 1E, S1D, and S1E, arrowheads) bones but also appeared to label bone-lining cells near the osteochondral junction of the growth plate (Figure 1F, arrows). Expression of vesicular ACh transporter (VAChT), which loads ACh into secretory organelles of cholinergic nerve terminals (Weihe et al., 1996), co-localized with ChAT-labeled bone-lining cells and cholinergic fibers associated with blood vessels (Figures 1G, 1H, S1F, and S1G). Vasoactive intestinal peptide (VIP), marking sympathetic cholinergic fibers in the periosteum (Asmus et al., 2000; Francis et al., 1997; Hohmann et al., 1986), followed a similar periosteal and perivascular staining pattern in cortical bone (Figure 1I). These data confirm the presence of cholinergic innervation in the periosteum and extend these findings to bone matrix and BM, where non-neural cholinergic osteolineage cells were additionally detected and characterized below (see Figure 4).

Binding of NRTN (Heuckeroth et al., 1999) to GFRα2 (Hiltunen and Airaksinen, 2004; Rossi et al., 1999) promotes the development and survival of cholinergic neurons (parasympathetic or sympathetic, but not noradrenergic). Therefore, we used mice lacking GFRα2 as a model of cholinergic neural deficiency. PGP9.5^+^, TUJ1^+^, VAChT^+^, or VIP^+^ neuronal patterns were reduced in *Gfra2*^*−/−*^ femurs (Figures 1J, 1K, S1H, and S1I). In contrast, TUJ1^+^ or VAChT^+^ cells lacking neural fiber morphology were preserved near the growth plate (Figures 1L, 1M, S1H, and S1I, arrows). Consistent with these confocal analyses (summarized in Figure 1N), unmyelinated—compatible with cholinergic—axons appeared reduced in *Gfra2*^*−/−*^ mice (Figures S1J and S1K). Therefore, loss of GFRα2 reduces autonomic cholinergic innervation in cortical bone but spares non-neuronal cholinergic cells near the growth plate.

### Sympathetic cholinergic nerve fibers in bone

**Figure 2:**
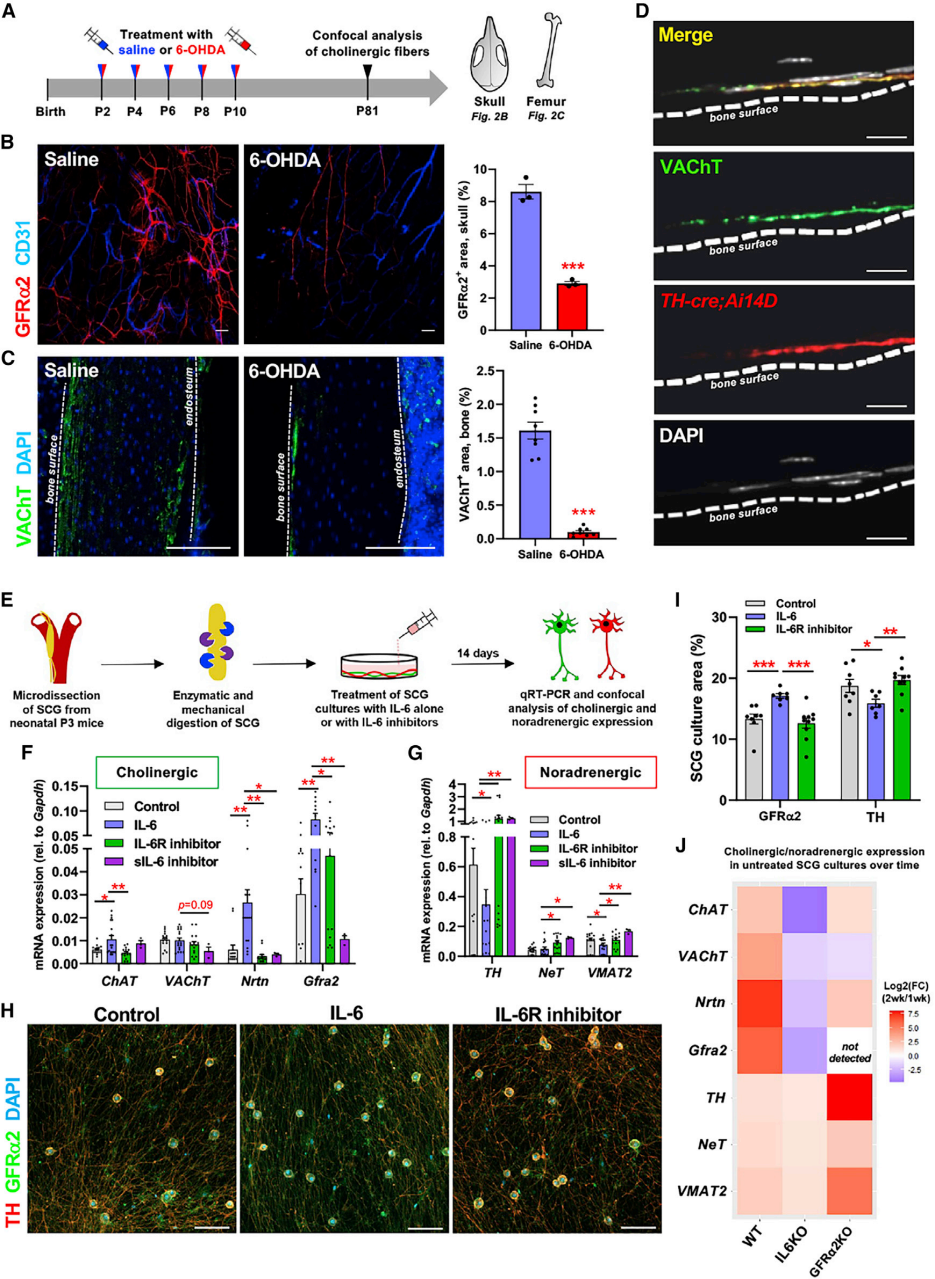
Interleukin-6 induces a cholinergic switch in sympathetic neurons (A) Schematic of neonatal sympathectomy and analysis at adulthood. (B and C) Immunofluorescence of (B) GFRα2^+^ or (C) VAChT^+^ cholinergic nerve fibers in skulls (B) and the cortical bone (C) of adult mice subjected to neonatal chemical sympathectomy (6-OHDA) or saline treatment, with quantification of cholinergic nerve fibers. See also Figures S2A–S2C. (D) Immunofluorescence of VAChT in genetically traced sympathetic nerve fibers from *TH-cre*;*Ai14D* bones. (E) Schematic of superior cervical ganglion (SCG) isolation and culture. (F and G) qRT-PCR analysis of (F) cholinergic and (G) noradrenergic gene expression from WT SCG cultures. (H and I) Immunofluorescence of (H) noradrenergic (TH) and cholinergic (GFRα2) markers in 14-day cultured WT SCGs and (I) quantification. (J) Heatmap depicting fold change in mRNA expression of cholinergic and noradrenergic genes from day 14 SCG cultures relative to day 7 SCG cultures (n= 3–5). See also Figures S3A–S3G. (C, D, and H) Nuclei were counterstained with DAPI. (B–D and H) Scale bars, 100 μm. (B, C, F, G, and I) Data are mean ± SEM. ^∗^p < 0.05, ^∗∗^p < 0.01, and ^∗∗∗^p < 0.001; ANOVA and pairwise comparisons.

A previous study suggested that cholinergic fibers innervating bone are parasympathetic based on retrograde tracing to thoracic and sacral spinal cord segments (Bajayo et al., 2012). However, a sympathetic origin has been proposed for sacral autonomic outflow (Espinosa-Medina et al., 2016). To clarify the origin of cholinergic fibers, neonatal mice were treated with 6-hydroxydopamine (6-OHDA) to ablate sympathetic fibers before the cholinergic switch during postnatal development (Figure 2A). At adulthood, similar reductions of noradrenergic (TH^+^) fibers and cholinergic (GFRα2^+^ or VAChT^+^) fibers (Hiltunen and Airaksinen, 2004) were observed in the femurs and skull bones of 6-OHDA-treated mice (Figures 2B, 2C, S2A, and S2C), suggesting a sympathetic origin of skeletal cholinergic fibers. For confirmation, we intercrossed *TH-cre* mice with *Ai14D* reporter mice and found that VAChT^+^ staining frequently co-localized with genetically traced sympathetic fibers near bone (Figure 2D). Furthermore, VAChT^+^ and GFRα2^+^ cholinergic axons traveled in the same nerve bundles as TH^+^ noradrenergic fibers, showing separation with successive branching (Figures S2D and S2E). Overall, these results support a sympathetic origin for skeletal cholinergic fibers.

### Sympathetic cholinergic fibers are preserved in bone lacking CNTF, CT-1, and LIF

Previous studies have shown that the IL-6 superfamily cytokines—such as leukemia inhibitory factor (LIF) (Rao and Landis, 1990; Yamamori et al., 1989), ciliary neurotrophic factor (CNTF) (Loy et al., 2011; Saadat et al., 1989), and cardiotrophin-1 (CT-1) (Habecker et al., 1997)—promote cholinergic gene expression *in vitro* (Ernsberger and Rohrer, 1999) but are not essential for the cholinergic switch *in vivo* (Francis et al., 1997; Habecker et al., 1997), suggesting redundancy or compensation. To test this hypothesis, we generated triple knockout (TKO) mice lacking CNTF, CT-1, and LIF. Notably, cholinergic innervation was unchanged in the skull or long bones of TKO mice (Figures S2F–S2H), prompting the search for an alternative factor triggering the cholinergic switch in bone.

### Interleukin-6 triggers a cholinergic switch in sympathetic neurons

IL-6 was an interesting candidate because—similar to CNTF, CT-1, and LIF—its signaling requires gp130 (Ip et al., 1992; Taga et al., 1989), which is essential for the cholinergic switch (Stanke et al., 2006) but also entails a unique co-receptor, IL-6R. Primary superior cervical ganglion (SCG) sympathetic neurons were treated with recombinant mouse (rm) IL-6 alone or in combination with inactivating antibodies against the mouse soluble IL-6 ligand (anti-mIL-6-IgG) or the human IL-6 receptor (tocilizumab). The expression of cholinergic and noradrenergic markers was measured after 14 days in culture (Figure 2E). rmIL-6 caused selective induction of cholinergic markers (Figure 2F) and downregulation of noradrenergic markers (Figure 2G). These effects were reversed by IL-6 inhibitors (Figures 2F and 2G), demonstrating specificity. Confocal analyses of rmIL-6-treated SCG cultures confirmed increased GFRα2^+^ (cholinergic) and reduced TH^+^ (noradrenergic) staining, while co-treatment with tocilizumab abrogated the cholinergic switch (Figures 2H and 2I).

WT SCG cultures showed endogenous IL-6 expression (Figures S3A and S3B) and spontaneous induction of cholinergic markers; in contrast, the cholinergic switch was nearly abrogated in *Il6*^*−/−*^ cultures (Figures 2J and S3C–S3E). These results demonstrate that IL-6 can induce a neuronal cholinergic switch *in vitro*. Contrarily, noradrenergic gene expression increased over time in untreated *Gfra2*^*−/−*^ SCG cultures (Figure 2J); however, *Il6* mRNA expression was normal (Figures S3A and S3B), suggesting an altered response to IL-6. Indeed, instead of inducing a cholinergic switch, rmIL-6 increased noradrenergic marker expression in *Gfra2*^*−/−*^ neurons (Figures S3F and S3G), likely due to different *cis*/*trans* IL-6 signaling: *cis*-signaling involves the natural binding of IL-6 to its receptor and subsequent gp130 activation, while *trans*-signaling results from the cleavage of IL-6R, producing a soluble IL-6R that can bind IL-6 and activate gp130 in other cells, leading to distinct differences in signal specificity, timing, amplification, and overall cellular phenotypes (Rose-John et al., 2017). *Gfra2*^*−/−*^ SCG neurons showed increased expression of TNFα-converting enzyme (TACE), one of the proteases responsible for the cleavage of membrane-bound IL-6R (Solomon et al., 2007) (Figure S3H), suggesting that resistance to cholinergic induction may be mediated through *trans*-IL-6-signaling. Supporting this possibility, TACE inhibition during the 1^st^ two postnatal weeks normalized cholinergic and noradrenergic nerve fibers in the bones of *Gfra2*^*−/−*^ mice (Figures S3I and S3J).

### Interleukin-6 promotes a sympathetic cholinergic switch in bone

**Figure 3:**
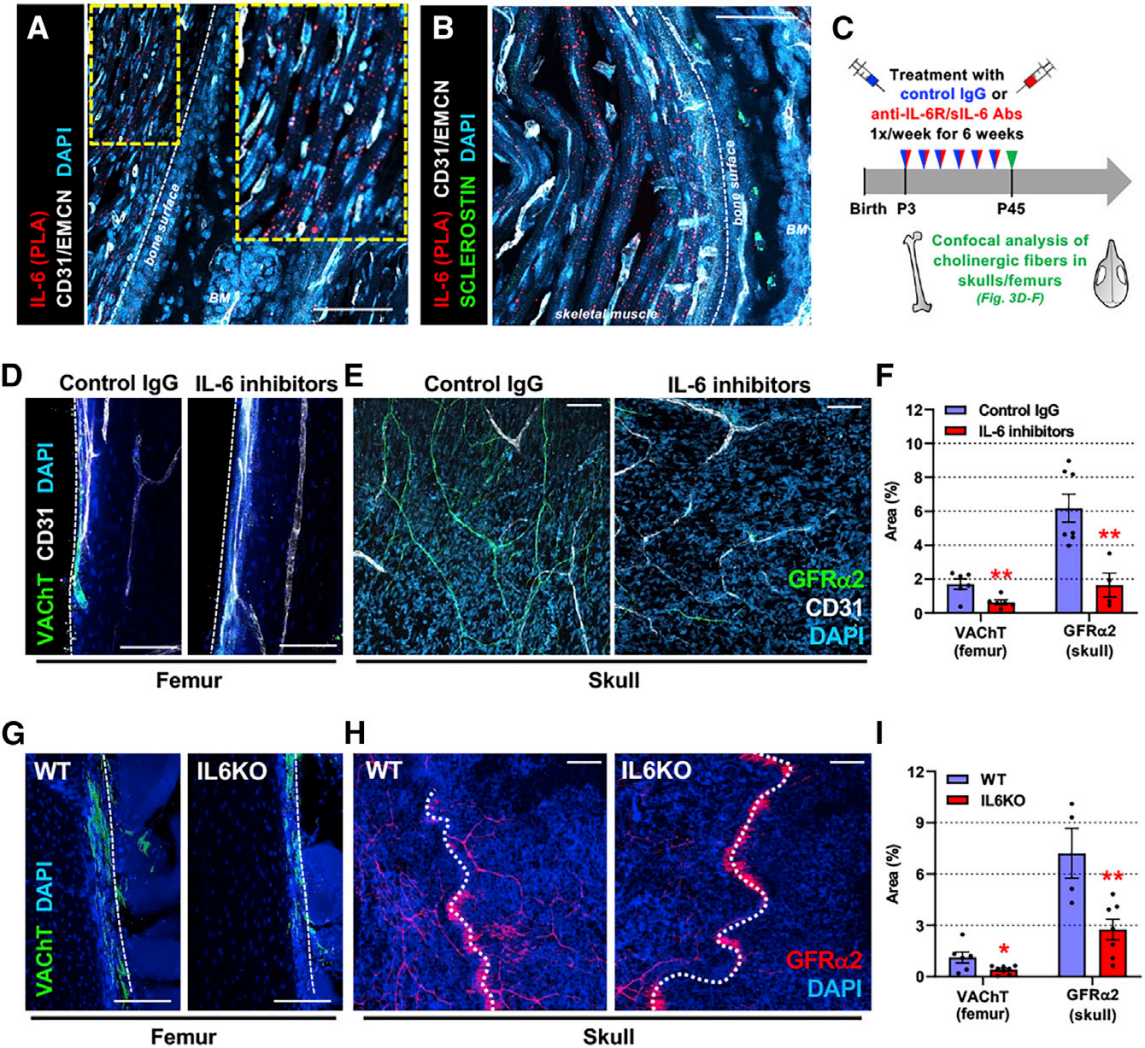
Interleukin-6 promotes postnatal development of skeletal sympathetic cholinergic innervation (A and B) Proximity ligation assay of IL-6 in BM section from postnatal day-3 developing femur. Scale bars, 100 μm (A) and 50 μm (B). (C) Schematic of *in vivo* IL-6 blockade. (D–F) Immunofluorescence of (D) VAChT^+^, or (E) GFRα2^+^ cholinergic nerve fibers (green) in (D) femoral cortical bone and (E) skulls from 6.5-week-old mice following IL-6 blockade, with (F) quantification of fiber area. Dashed lines indicate bone surface. (G–I) Immunofluorescence of (G) VAChT^+^ or (H) GFRα2^+^ cholinergic nerve fibers in (G) femoral cortical bone and (H) skulls from male IL-6 KO mice, with (I) quantification of fiber area. Dashed lines indicate cranial sutures. (D, E, G, and H) Scale bars, 100 μm. Nuclei were counterstained with DAPI (blue). (F and I) Data are mean ± SEM, ^∗^p < 0.05, ^∗∗^p < 0.01, unpaired two-tailed t test.

Because IL-6 enhances the cholinergic phenotype in developing sympathetic neurons *in vitro*, we examined IL-6’s source and potential to drive the cholinergic switch *in vivo*. A proximity ligation assay of postnatal day 3 developing limbs showed high IL-6 near the periosteum, mainly in adjacent skeletal muscle (Figures 3A and 3B). Therefore, we treated mice with IL-6 inhibitors or control IgG weekly during the first 6 postnatal weeks (Figure 3C). Femurs and skulls of mice treated with IL-6 inhibitors showed a normal presence of TH^+^ noradrenergic nerve fibers (Figures S3K and S3L), but a 3- to 4-fold reduction in VAChT^+^ or GFRα2^+^ cholinergic fibers (Figures 3D–3F). Similarly, *Il6*^*−/−*^ mice exhibited ∼3-fold-reduced cholinergic fiber density in femurs and skulls (Figures 3G–3I), suggesting that IL-6 can promote the cholinergic phenotype *in vivo*.

### Osteolineage cells contribute to the non-neuronal cholinergic system

**Figure 4:**
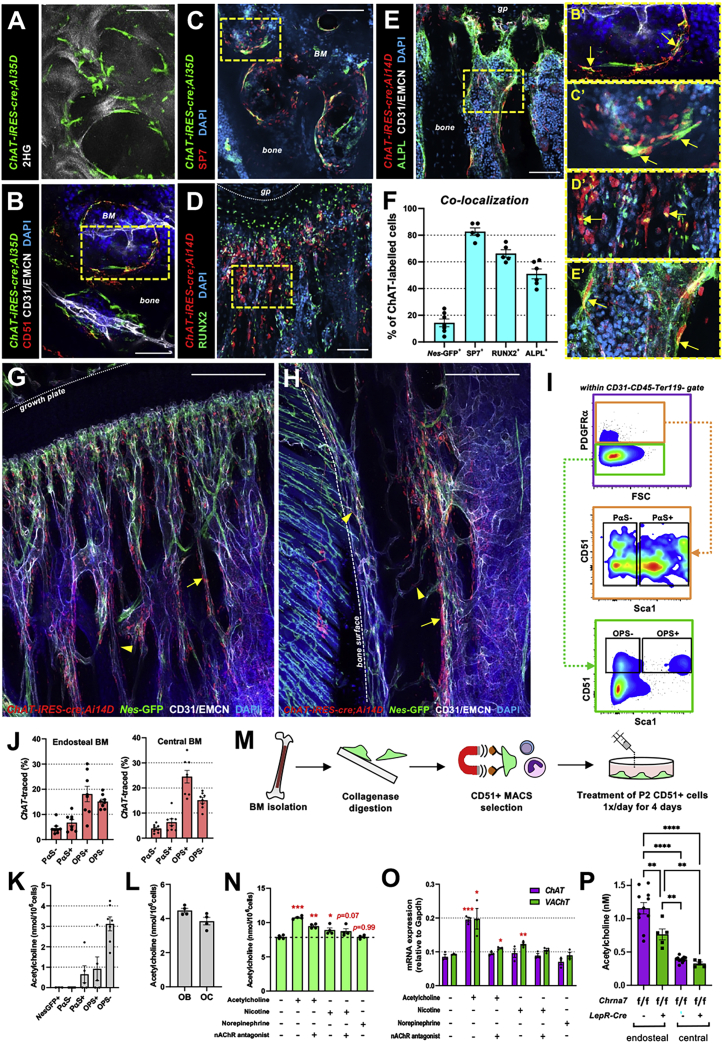
Osteolineage cells contribute to the non-neuronal cholinergic system (A–E) Immunofluorescence of cholinergic bone-lining cells near the growth plate (gp) in (A–C) *ChAT-IRES-cre;Ai35D* and (D and E) *ChAT-IRES-cre;Ai14D* mice with co-staining of osteolineage markers. Arrows depict co-localization in high-magnification insets (Bʹ–Eʺ). 2HG, 2^nd^ harmonic generation imaging of collagen. See also Figures S4A and S4B. (F) Quantification of co-localization of ChAT-labeled cells. (G and H) Immunofluorescence of cholinergic bone-lining cells near the (G) growth plate and (H) endosteal regions of *ChAT-IRES-cre;Ai14D;Nes-GFP* tibias. Arrows depict nerve fibers. Arrowheads depict osteolineage cells. See also Figures S4C and S4D. (A–E) Scale bars, 100 μm (A and B) and 200 μm (G and H). (I) Flow cytometry gating strategy for analysis of CD31^−^CD45^−^Ter119^−^PDGFRα^+^Sca1^−^ (PαS^−^) cells, CD31^−^CD45^−^Ter119^−^PDGFRα^+^Sca1^+^ (PαS^+^) cells, CD31^−^CD45^−^Ter119^−^PDGFRα^−^CD51^+^Sca1^+^ (OPS^+^) cells, and CD31^−^CD45^−^Ter119^−^PDGFRα^−^CD51^+^Sca1^−^ (OPS^−^) cells. (J) Frequency of *ChAT-IRES-cre*-traced osteolineage cells in endosteal or central BM. See also Figures S4E and S4F. (K) Acetylcholine content in osteolineage cells from WT and *Nes-GFP* mice. (L) Acetylcholine content in primary WT osteoblasts (OB) and osteocytes (OC) from digested WT bone fragments. (M) Schematic of CD51^+^ osteolineage cell isolation and culture. (N and O) Acetylcholine content (N) and qRT-PCR analysis (O) of cultured CD51^+^ osteolineage cells. (P) Acetylcholine content from endosteal and central BM serum of mice lacking an α7 nicotinic receptor in *LepR-cre* targeted niche cells 1 month after bone marrow transplantation. (F and J–P) Data are mean ± SEM, ^∗^p < 0.05, ^∗∗^p < 0.01, ^∗∗∗^p < 0.001, and ^∗∗∗∗^p < 0.0001; unpaired two-tailed t test (F and J–O) or ANOVA and pairwise comparisons (P).

Visualization of bone collagen in *ChAT-iRES-cre*;*Ai35D* mice revealed ChAT-traced cells lining the bone surface in the BM adjacent to the epiphyseal plate (Figures 4A, S4A, and S4B). The majority of ChAT-traced growth plate cells were osteogenic since they co-expressed the markers CD51 (Green et al., 2021; Matic et al., 2016; Noll et al., 2014), the transcription factor osterix (SP7), alkaline phosphatase (ALPL), or RUNX2 (Figures 4B–4F). In *ChAT-iRES-cre*;*Ai14D* reporter mice intercrossed with *Nes-gfp* mice, ChAT-traced neuronal patterns (arrowheads) or bone-lining cells (arrows) were very close to, but distinct from, perivascular *Nes*-GFP^+^ SSC-enriched cells (Figures 4G, 4H, S4C, and S4D). The frequency of ChAT-traced cells increased with osteogenic commitment, as labeling was higher in downstream osteoprogenitors than in more primitive PDGFRα^+^ cells (Gulati et al., 2018; Matic et al., 2016; Morikawa et al., 2009; Noll et al., 2014; Pinho et al., 2013) (Figures 4I, 4J, S4E, and S4F). Consistently, ACh content was highest in osteoprogenitor cells (Figure 4K). Enzymatic digestion of bone fragments (Asada et al., 2013; Stern et al., 2012) confirmed ACh content in primary osteoblasts (pOBs) and bone-embedded osteocytes (Figure 4L). ACh concentration was reduced in the BM serum—but not the osteolineage cells—of *Gfra2*^*−/−*^ mice (Figure S4G), consistent with normal VAChT expression in growth plate cells (see Figures 1L and 1M). These data confirm the specific cholinergic neural deficiency in *Gfra2*^*−/−*^ mice and identify osteolineage cells as a component of the non-neuronal cholinergic system in BM.

Given that cholinergic innervation is enriched at periosteal and cortical sites but regulates hematopoietic cells deeper in the BM (Fielding et al., 2022; García-García et al., 2019), we hypothesized that osteolineage cells containing ACh could transmit and amplify the cholinergic signal in BM. CD51^+^ osteolineage cells showed higher expression of nicotinic ACh receptors compared with CD51^*−*^ cells (Figures S4H and S4I). Therefore, we treated CD51^+^ cells with cholinergic agonists, antagonists, or control medium (Figure 4M). ACh or nicotine doubled *ChAT* and *VAChT* mRNA expression and increased ACh content in cultured CD51^+^ cells; these effects were attenuated with the nicotinic antagonist, hexamethonium (Figures 4N and 4O). In a separate study, we found that cholinergic signals increase after myeloablation or irradiation and preserve hematopoietic stem cell quiescence after transplantation via an α7 nicotinic receptor in niche cells (Fielding et al., 2022). Four weeks after lethal irradiation and transplantation of BM cells, ACh content was reduced in the endosteal (not central) BM of recipient mice lacking an α7 nicotinic receptor in Leptin-receptor-Cre-targeted niche cells (Ding et al., 2012), which largely overlap with *Nes*-GFP^+^ SSC-enriched cells (Mende et al., 2019; Méndez-Ferrer, 2019) (Figure 4P). Therefore, osteolineage cells may transmit and amplify cholinergic neural signals in bone and BM.

### Osteopenia and reduced bone formation in *Gfra2*^*−/−*^ mice

**Figure 5:**
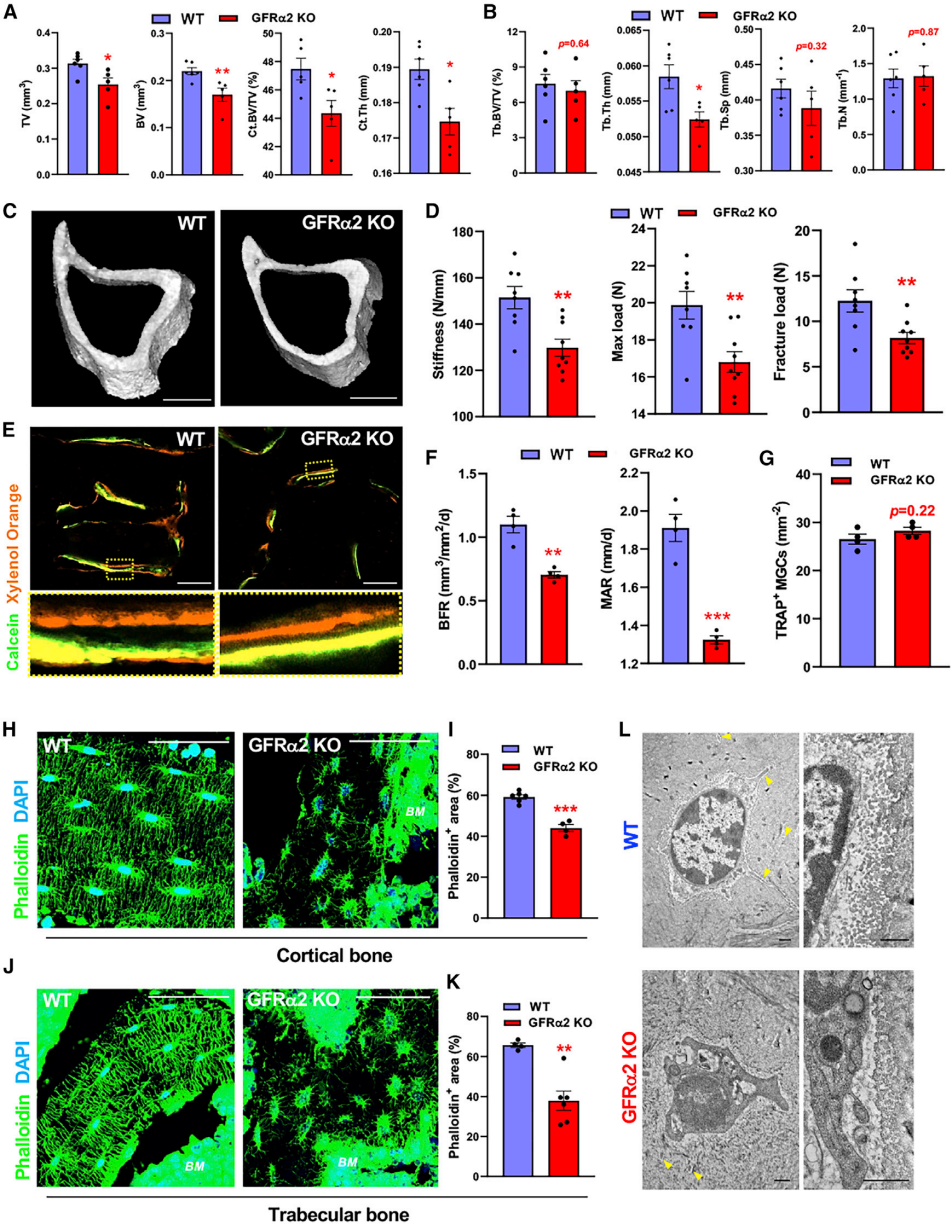
GFRα2 loss causes osteopenia and osteocyte degeneration (A and B) Quantitative μCT analysis of 3D cortical (A) and trabecular (B) bone parameters in WT or GFRα2 KO female tibias: tissue volume (TV), bone volume (BV), cortical bone volume fraction (Ct.BV/TV), cortical thickness (Ct.Th), trabecular bone volume fraction (Tb.BV/TV), trabecular thickness (Tb.Th), trabecular separation (Tb.Sp), and trabecular number (Tb.N). See also Figures S5A and S5B. (C) 3D rendering of proximal mid-tibial diaphysis from WT or GFRα2 KO female mice. Scale bars, 1 mm. (D) Three-point bend testing of tibias from WT or GFRα2 KO female mice. See also Figure S5E. (E and F) Immunofluorescence of trabecular bone from WT or GFRα2 KO female mice injected with calcein and xylenol orange (E) with quantification of bone formation rate (BFR) and mineral apposition rate (MAR) (F). Scale bars, 50 μm. See also Figure S5F. (G) Quantification of tartrate-resistant acid phosphatase (TRAP^+^) multinucleated giant cells (MGCs) from WT or GFRα2 KO BM sections. See also Figure S5G. (H–K) Phalloidin staining (H and J, green) and quantification (I and K) of osteocytes embedded in (H and I) cortical bone or (J and K) trabecular bone from WT or GFRα2 KO mice. Nuclei were counterstained with DAPI (blue). Scale bars, 50 μm. (L) Transmission electron micrographs of osteocytes (left) and surrounding collagen matrix (right) from WT or GFRα2 KO humeri. Arrowheads depict osteocyte cell processes. Scale bars, 500 nm. See also Figures S6C and S6D. (A, B, D, F, G, I, and K) Data are mean ± SEM, ^∗^p < 0.05, ^∗∗^p < 0.01, ^∗∗∗^p < 0.001, unpaired two-tailed t test.

Since cholinergic activity promotes bone mass accrual (Bajayo et al., 2012; Shi et al., 2010), we asked whether cholinergic neural deficiency might compromise skeletogenesis or skeletal turnover. Cortical morphometry of tibias from *Gfra2*^*−/−*^ females showed reduced cortical bone size, volume, volume fraction, cortical bone thickness, and trabecular thickness, while trabecular separation and number remained unchanged (Figures 5A–5C). *Gfra2*^*−/−*^ male mice exhibited a milder phenotype with a trend toward a decrease in bone volume and thickness, which inversely correlated with cholinergic nerve fibers in cortical bone (Figures S5A–S5C). This suggests gender-specific bone phenotypes, as shown for *CNTF*^*−/−*^ mice lacking another gp130 ligand (McGregor et al., 2010). Cranial sutures were markedly expanded and skulls appeared flatter in *Gfra2*^*−/−*^ males (Figure S5D). Skeletal parameters reduced in *Gfra2*^*−/−*^ mice did not correlate with the expectedly reduced body weight of these mice (data not shown), uncoupling nutrition defects (McDonagh et al., 2007; Rossi et al., 1999, 2003) from the skeletal phenotypes. Notably, three-point bend tests showed reduced stiffness and strength in both female and male *Gfra2*^*−/−*^ tibias (Figures 5D and S5E).

The bone formation rate (BFR) and mineral apposition rate (MAR) were reduced in *Gfra2*^*−/−*^ mice (Figures 5E, 5F, and S5F); in contrast, tartrate-resistant acid phosphatase (TRAP^+^) osteoclasts and bone resorption markers were unchanged (Figures 5G and S5G–S5I). These results imply functional alterations of bone-forming (rather than bone-resorbing) cells. Using flow cytometry, PDGFRα^+^ cells were reduced in endosteal *Gfra2*^*−/−*^ BM, likely leading to their compensatory proliferation and increased osteoprogenitors in central BM (Figures S5J–S5O). Consistently with the immunophenotypic analysis, *Gfra2*^*−/−*^ BMSCs generated less self-renewing mesenchymal spheres (mesenspheres) and more osteoblastic colonies (CFU-OBs) (Figures S5P and S5Q).

### GFRα2 signaling maintains osteocyte connectivity and survival

**Figure 6:**
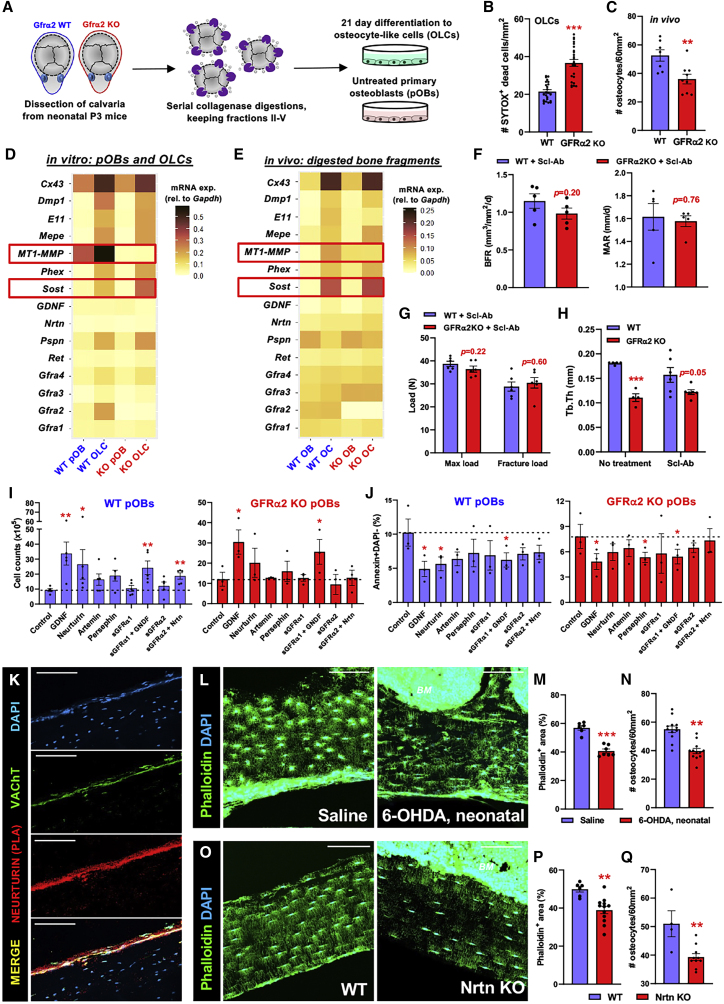
GFRα2 signaling maintains osteocyte connectivity and survival (A) Schematic of primary calvarial osteoblast (pOB) isolation and differentiation into osteocyte-like cells (OLCs). (B) Quantitative analysis of SYTOX^+^ dead cells in d21 WT or GFRα2 KO OLCs. (C) Number of osteocytes quantified from low-magnification phalloidin-stained cortical bone sections. See also Figure S6A. (D and E) Heatmap depicting mRNA expression from (D) calvarial pOBs and differentiated OLCs (n = 3–5) or (E) primary osteoblasts (OB) and osteocytes (OC) from digested WT or GFRα2 KO femur fragments (n = 6). (F–H) Analysis of dynamic histomorphometry (F, femurs), three-point bend (G, tibias), and trabecular thickness (Tb.Th, tibias) in male WT or GFRα2 KO mice subjected to treadmill exercise (5× per week) with s.c. treatment of Scl-Ab r13c7 (1 per week) for 5 weeks. (I and J) Cell numbers (I) and frequency of apoptotic (J) pOBs after 4-day treatment with GDNF-family ligands and soluble receptors. (K) Proximity ligation assay of neurturin in BM section from WT femur, with co-staining for VAChT^+^ cholinergic nerve fibers. (L–Q) Fluorescence (L and O, green) and quantification of phalloidin^+^ (M and P) osteocytes (N and Q) from adult WT mice subjected to neonatal chemical sympathectomy (6-OHDA; L–N), or WT or neurturin (Nrtn) KO mice (O–Q). (K, L, and O) Scale bars, 100 μm. Nuclei were counterstained with DAPI (blue). (B, C, F–J, M, N, P, and Q) Data are mean ± SEM, ^∗^p < 0.05, ^∗∗^p < 0.01, ^∗∗∗^p < 0.001, unpaired two-tailed t test.

The persistent osteopenia and reduced bone formation in *Gfra2*^*−/−*^ mice despite the increased osteoprogenitors suggested a defect in the orchestration of surface bone formation, which is normally achieved through the fine control of OB activity and recruitment by the network of mineral-embedded osteocytes (retired OBs); the osteocyte syncytium fulfills this role in controlling surface activity via connected dendrites networked within billions of fine canaliculi (Robling and Bonewald, 2020). In *Gfra2*^*−/−*^ mice, we observed grossly abnormal osteocyte morphology, showing large spherical or flattened cell bodies and reduced dendrites (Figures 5H–5K, S6A, and S6B). Transmission electron microscopy (TEM) confirmed reduced branching in *Gfra2*^*−/−*^ osteocytes and revealed membrane blebbing, abundant autophagosomes, and reduced lacunar space (Figures 5L, S6C, and S6D), suggesting osteocyte degeneration and impaired collagen cleavage. Osteocyte-like cells (OLCs) differentiated from *Gfra2*^*−/−*^ pOBs showed decreased survival, explaining ∼30% reduced osteocytes in *Gfra2*^*−/−*^ femurs (Figures 6A–6C).

To investigate GFRα2 signaling, we profiled the GDNF family of ligands and receptors in pOBs, OLCs, and primary osteolineage cells. *Gfra2* and related ligands and receptors were expressed in WT osteolineage cells, while *Gfra2* mRNA expression increased following osteogenic differentiation, and *Gfra2*^*−/−*^ osteocytes showed decreased expression of *Mt1-Mmp*—a membrane-anchored proteinase required for collagen cleavage and osteocyte branching (Holmbeck et al., 2005) and increased mRNA expression of sclerostin (*Sost*)—an inhibitor of Wnt signaling and bone formation secreted by osteocytes (Holdsworth et al., 2019) (Figures 6D and 6E). Furthermore, *Gfra2*^*−/−*^ osteocytes showed high sclerostin protein levels, which were resistant to their normal repression by mechanical loading (treadmill exercise) (Figure S6E). Mechanistically, sclerostin inhibition in exercised *Gfra2*^*−/−*^ mice normalized BFR, strength, and trabecular thickness (Figures 6F–6H), highlighting the relevance of sclerostin in the osteopenia of *Gfra2*^*−/−*^ mice. Skeletal responses of WT mice to sclerostin blockade were as expected (Holdsworth et al., 2019).

Because osteocytes express *Gfra2*, and *Gfra2*^*−/−*^ osteocytes exhibit survival defects, we next examined the trophic effects of GDNF-related ligands and/or soluble receptors *in vitro*. Treatment with GDNF or NRTN, alone or combined with their soluble receptors, improved growth and survival in WT pOBs, while NRTN’s trophic effect was reduced in *Gfra2*^*−/−*^ pOBs (Figures 6I and 6J). MLO-Y4 OLCs (Kato et al., 1997) similarly exhibited reduced apoptosis upon sGFRα1/2 treatment (Figures S6F and S6G), suggesting the trophic effect of NRTN-GFRα2 signaling in osteocytes.

### Cholinergic fibers in bone maintain osteocyte survival and connectivity

Proximity ligation assay showed the highest NRTN expression among cholinergic fibers in bone (Figure 6K), suggesting that these fibers can activate NRTN co-receptor RET signaling in osteocytes and their lack may cause osteocyte degeneration in GFRα2-expressing osteocytes *in vivo*. Indeed, neonatal sympathectomy (to ablate adult peripheral skeletal cholinergic fibers) similarly reduced adult osteocyte number and dendritic branching (Figures 6L–6N), which was phenocopied in *Nrtn*^*−/−*^ mice (Figures 6O–6Q), suggesting that NRTN-GFRα2 signaling promotes osteocyte survival. Since 6-OHDA treatment in neonates (before the cholinergic switch) ablates adult sympathetic cholinergic and noradrenergic fibers, for comparison we administered 6-OHDA in adult mice, selectively ablating noradrenergic (but not cholinergic) fibers (Figure S6H). Contrasting neonatal treatment, adult 6-OHDA treatment did not affect osteocyte morphology, branching, or numbers (Figures S6I–S6K). Therefore, lack of peripheral sympathetic cholinergic fibers, or NRTN, in mice with GFRα2-competent osteocytes phenocopies the osteocyte defects associated with global GFRα2 deficiency, suggesting that this neuro-osteocyte interface preserves the osteocyte network.

### Moderate exercise increases bone cholinergic innervation through interleukin-6

Having demonstrated osteopenia caused by deficient cholinergic innervation of bone, we investigated the possible bone-anabolic effects of increased sympathetic cholinergic activity. Since physical activity during adolescence largely influences peak bone mass (Weaver et al., 2016), moderate exercise in young rodents was selected as a gain-of-function model. Wistar rats (used to confirm the skeletal sympathetic cholinergic innervation in different species) underwent early postnatal sympathectomy or vehicle treatment, followed by treadmill running (Figure S7A). Consistent with findings in mice, sympathectomy ablated both TH^+^ noradrenergic fibers and GFRα2^+^ cholinergic fibers in femurs of Wistar rats (Figures S7B–S7E), confirming a sympathetic origin. Importantly, moderate exercise nearly tripled GFRα2^+^ cholinergic fibers in femoral BM (Figures S7D and S7E), suggesting that physical activity increases skeletal cholinergic innervation. This correlated with the expectedly increased trabecular (not cortical) bone volume fraction, trabecular number, and reduced trabecular separation in exercised rodents (Berman et al., 2019); importantly, skeletal adaptations to moderate exercise were abrogated by early postnatal sympathectomy (Figures S7F–S7I). These results suggest that skeletal sympathetic cholinergic innervation increases during exercise to promote bone formation.

**Figure 7:**
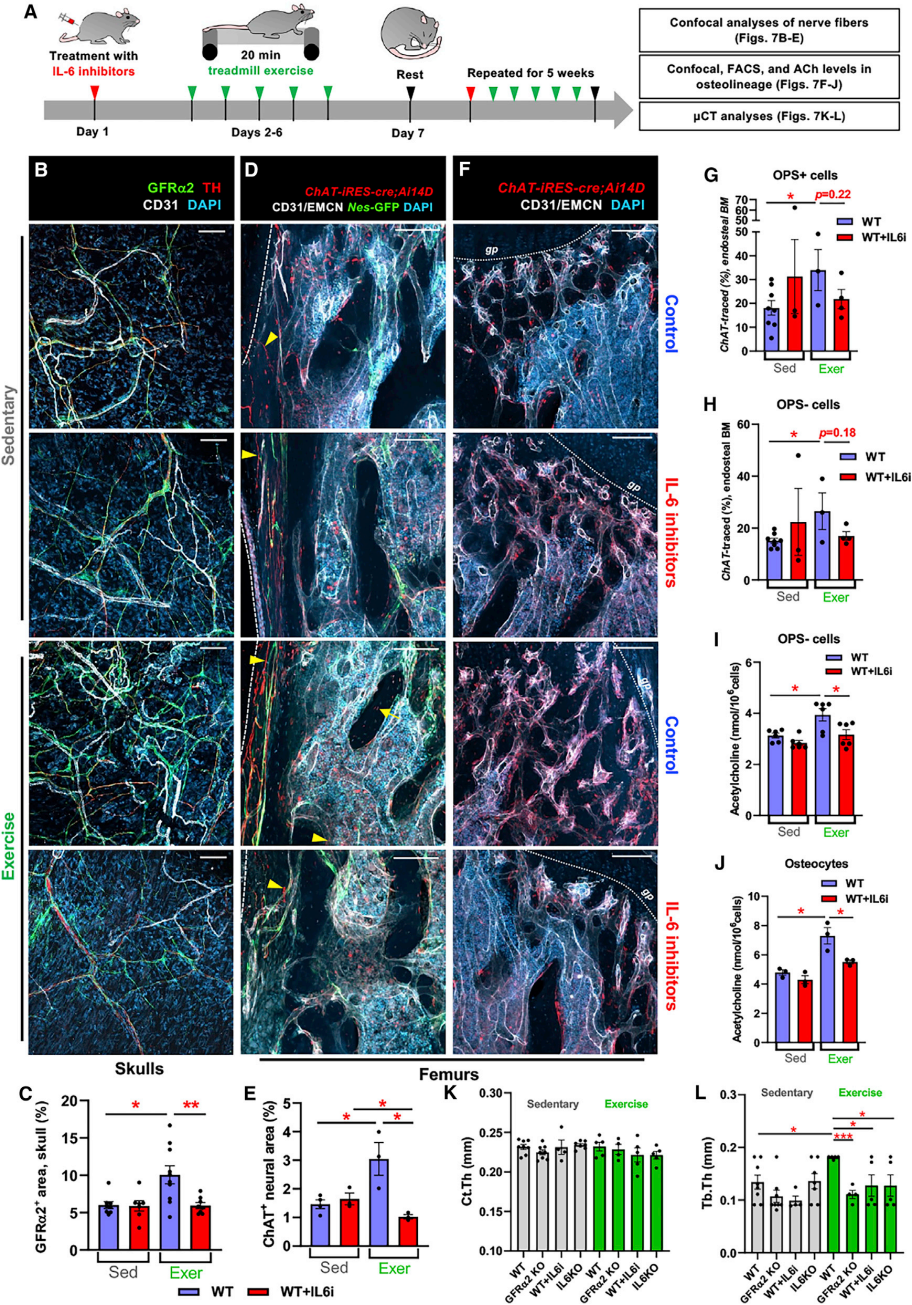
Moderate exercise increases bone cholinergic innervation through interleukin-6 (A) Schematic of moderate exercise protocol and analysis. (B–J) Characterization of cholinergic cells in sedentary or exercised (B, C, I, and J) WT mice or (D–H) *ChAT-IRES-cre;Ai14D;Nes-GFP* mice treated with IL-6 inhibitors or control IgG. (B and C) Immunofluorescence (B) and quantification (C) of GFRα2^+^ cholinergic fibers in skulls. Tyrosine hydroxylase (TH)^+^ noradrenergic fibers are also shown. (D and E) Immunofluorescence (D) and quantification (E) of genetically marked cholinergic nerve fibers (arrowheads). Arrow depicts ChAT^+^ osteocyte. (F–H) Immunofluorescence (F) and flow cytometric quantification (G and H) of genetically traced cholinergic osteolineage cells including (G) CD31^−^CD45^−^Ter119^−^PDGFRα^−^CD51^+^Sca1^+^ OPS+ cells and (H) CD31^−^CD45^−^Ter119^−^PDGFRα^−^CD51^+^Sca1^−^ OPS− cells. (B, D, and F) Nuclei were counterstained with DAPI (blue). Scale bars, 100 μm. (I and J) Acetylcholine content in (I) OPS^−^ cells and (J) osteocytes. (K and L) Quantitative μCT analysis of (K) cortical thickness (Ct.Th) and (L) trabecular thickness (Tb.Th) in sedentary or exercised male control WT mice, WT mice treated with IL-6 inhibitors, GFRα2 KO mice, or IL-6 KO mice. (C, E, and G–L) Data are mean ± SEM, ^∗^p < 0.05, ^∗∗^p < 0.01, ^∗∗∗^p < 0.001, unpaired two-tailed t test.

Skeletal muscle-derived IL-6 regulates bone remodeling during exercise (Chowdhury et al., 2020). Since IL-6 can drive the cholinergic switch (see Figures 2, 3, S2, and S3), we wondered whether IL-6 could boost cholinergic activity to facilitate skeletal adaptation to exercise in young mice. Cholinergic fiber density was doubled in skulls of exercised mice, but not after IL-6 blockade (Figures 7A–7C), suggesting a role for circulating IL-6. Similar results were obtained in *ChAT-IRES-cre;Ai14D;Nes-gfp* mice; moderate exercise increased cholinergic fiber density near perivascular *Nes*-GFP^+^ SSC-enriched cells, but not after IL-6 blockade (Figures 7D and 7E).

Since our *in vitro* studies showed that ACh stimulation can increase ACh content in osteolineage cells (see Figures 4N and 4O), we asked whether exercise-induced sympathetic cholinergic activity propagates to osteolineage cells *in vivo*. Supporting this concept, *ChAT*-traced osteoprogenitors expanded (Figures 7F–7H) and ACh concentration (Figures 7I and 7J) increased in osteoprogenitors and osteocytes from exercised mice, but not after IL-6 blockade, matching the cholinergic neural response (see Figures 7B–7E) and further suggesting impaired cholinergic propagation in bone-forming cells. Importantly, consistent with results in the rat model, moderate exercise increased trabecular thickness in WT mice, but not in *Gfra2*^*−/−*^ mice or WT mice with IL-6 blockade (Figures 7K and 7L). These results suggest that IL-6 not only drives the cholinergic switch during postnatal development, but also serves to strengthen the cholinergic regulation of the skeleton in response to physical activity during adolescence.

## Discussion

Here, we characterized the neuronal and non-neuronal cholinergic system in bone. We found that skeletal sympathetic cholinergic nerve fibers, which are induced by IL-6, preserve osteocyte survival and function through the NRTN-GFRα2 neurotrophic axis during postnatal development and physical activity in adolescence. These conclusions are supported by: (1) cholinergic nerve fibers being the main source of NRTN near bone (Figure 6K); (2) NRTN directly promoting osteocyte survival (Figures 6I and 6J) in GFRα2- and RET-expressing osteocytes (Figures 6D and 6E); (3) treatment with GDNF or NRTN improving growth and survival in WT pOBs, while NRTN’s trophic effect is reduced in *Gfra2*^*−/−*^ pOBs (Figures 6I and 6J); (4) MLO-Y4 OLCs (Kato et al., 1997) similarly exhibiting reduced apoptosis upon GFR treatment (Figures S6F and S6G); (5) osteocyte numbers being reduced and atrophic in *Nrtn* KO mice (Figures 6O–6Q) or after neonatal sympathectomy of cholinergic fibers (Figures 6L–6N), but not after adult sympathectomy of noradrenergic fibers (Figures S6H–S6K); (6) bone adaptation to moderate exercise being impaired in cholinergic-neural-deficient mice (Figure 7L) or in rats after neonatal sympathectomy of cholinergic fibers (Figures S7F–S7I); (7) deficient bone-anabolic responses in cholinergic-neural-deficient mice, explained by the incapacity of osteocytes to repress sclerostin, which is a key inhibitor of bone-anabolic Wnt signaling (Figures 6D, 6E, and S6E); and (8) the key role of deregulated sclerostin in the absence of sympathetic cholinergic fibers, which is demonstrated by the rescue of osteopenia and bone strength in GFRα2 KO mice treated with sclerostin-blocking antibody (Figures 6F–6H).

Our study confirms the presence of cholinergic innervation in the periosteum and extends these findings to bone matrix and BM near the epiphyseal growth plate. Furthermore, osteolineage cells emerge as an additional component of the non-neuronal cholinergic system in bone. Treatment with ACh increases cholinergic markers and ACh content in CD51^+^ osteolineage cells, but not after nicotinic receptor blockade, suggesting that cholinergic neural signals are relayed to osteolineage cholinergic cells. Supporting this possibility, ACh levels were higher in the endosteal BM of chimeric mice and were specifically reduced in the endosteal BM upon α7 nicotinic deletion in *LepR-Cre*-targeted cells. These results suggest that cholinergic neural signals are relayed to bone-forming cells.

Since central or peripheral cholinergic activity promotes bone mass accrual (Bajayo et al., 2012; Shi et al., 2010), we asked whether the lack of skeletal sympathetic cholinergic fibers might compromise skeletogenesis or skeletal turnover. Long bones from *Gfra2*^*−/−*^ mice show normal bone-resorbing parameters but reduced SSCs and bone formation, leading to decreased bone mass and strength, enlarged cranial sutures, and flatter skulls. Impaired bone-anabolic response appears to result from structural and functional alterations in bone-embedded osteocytes. The osteocyte network plays a key role in orchestrating bone remodeling and IL-6- and Wnt-dependent bone-anabolic responses to mechanical loading during physical activity (Robling and Bonewald, 2020). Mechanistically, *Gfra2*^*−/−*^ osteocytes overproduce the Wnt inhibitor sclerostin, and sclerostin blockade rescues many of the histomorphometric defects. Peripheral sympathectomy at neonatal stage (ablating cholinergic fibers)—but not at adult stage (ablating only noradrenergic fibers)—recapitulates the osteocyte defects of *Gfra2*^*−/−*^ mice. Therefore, we conclude that skeletal sympathetic cholinergic fibers have bone-anabolic effects complementary to those of central cholinergic inhibition of sympathetic tone (Shi et al, 2010). GFRα2^+^ fibers are also detected in the BM of Wistar rats and resemble cholinergic fibers recently reported in human bone (Courties et al., 2020), suggesting interspecies conservation.

Both in mice and Wistar rats, moderate exercise doubles skeletal cholinergic fibers, correlated with increased trabecular bone. However, cholinergic fiber induction and increased ACh concentration in osteoprogenitors are blunted by IL-6 blockade in mice. Furthermore, skeletal adaptation to moderate exercise is severely compromised by early postnatal sympathectomy in rats. Therefore, we conclude that IL-6-driven cholinergic signals are required for the skeletal adaptation to exercise. In humans, IL-6 gene variants have been associated with osteoporosis and osteopenia (Ota et al., 1999, 2001). In our study, the bone-anabolic effects of cholinergic signals appear to involve *cis*-IL6-signaling (instead of *trans*-signaling, which may have opposite effects) (Rose-John et al., 2017). The conclusions are consistent with findings in menopause-related osteoporosis, where excessive *trans*- (not *cis*-) IL-6 signaling causes loss of trabecular bone (Lazzaro et al., 2018; Sims, 2021), mirroring the gain of trabecular bone through *cis*-IL6-induced cholinergic signals.

Genetic lineage tracing and early postnatal sympathectomy in rodents reveal a sympathetic origin of skeletal cholinergic nerve fibers. These axons appear to travel in the same nerve bundles as noradrenergic nerve fibers before branching, suggesting potential inhibitory feedback loops between these fibers as shown in other organs/tissues such as the pancreas (Benthem et al., 2001), eyelid smooth muscle (Beauregard and Smith, 1994), trachea (Pendry and Maclagan, 1991), and heart (Azevedo and Parker, 1999; Gavioli et al., 2014; Hasan and Smith, 2009; Miyashita et al., 1999; Smith-White et al., 1999). Moreover, *Gfra2*^*−/−*^ mice exhibit increased sympathetic noradrenergic innervation in the BM (García-García et al., 2019), similarly supporting putative inhibitory feedback loops. While noradrenergic fibers are found throughout the BM, cholinergic fibers are preferentially located in cortical bone with sprouting branches localized in trabecular BM. Although our data strongly argues for a spatial segregation of noradrenergic and cholinergic axons, we cannot exclude the possibility that some nerve fibers might have mixed and/or highly dynamic properties. The sympathetic SCG contains neurons with combined noradrenergic and cholinergic properties (Furshpan et al., 1986; Landis, 1976), and different neurotrophic factors can rapidly affect neurotransmitter synthesis, storage, release, and uptake (Luther and Birren, 2009; Yang et al., 2002).

Cholinergic signals are propagated to the BM through bone-lining osteoprogenitors, which transmit and amplify the cholinergic signal to the BM matrix, regulate the migration of hematopoietic stem cells and leukocytes (García-García et al., 2019), and preserve hematopoietic stem cell quiescence during hematopoietic regeneration (Fielding et al., 2022). These results add to the osteocyte network’s regulatory role in propagating noradrenergic signals to BM (Asada et al., 2013). Finally, since increased IL-6 during moderate exercise expands bone-anabolic cholinergic fibers, the achievement of peak bone mass, which is an important predictor of osteoporosis in late adulthood, may be mediated at least in part by the sympathetic cholinergic system and may represent a drug-able target for maintenance of peak bone mass.

### Limitations of the study

Although the results show NRTN-GFRα2 in the maintenance of the neuro-osteocyte interface, other signals might also contribute. Similarly, while deregulated sclerostin expression in osteocytes explains many skeletal phenotypes, other mechanisms and cell types regulated by cholinergic signals might participate in the complex interplay identified here between the skeletal and peripheral neural systems.

## STAR★Methods

### Key resources table

**Table:** 

REAGENT or RESOURCE	SOURCE	IDENTIFIER
**Antibodies**		

Biotin anti-mouse CD51 antibody	BioLegend	Cat. No. 104104; RRID:AB_313073
Rabbit anti-tyrosine hydroxylase	Merck	Cat. No. AB152; RRID:AB_390204
Chicken anti-GFP antibody	Aves Labs	Cat. No. GFP-1020; RRID:AB_10000240
Rabbit anti-GFP antibody	Abcam	Cat. No. ab290; RRID:AB_303395
Living Colors DsRed polyclonal antibody	Takara/Clontech	Cat. No. 632496; RRID:AB_10013483
Rat anti-endomucin (V.7C7) antibody	Santa Cruz	Cat. No. sc-65495; RRID:AB_2100037
α-Smooth Muscle Actin-Cy3 antibody	Sigma-Aldrich	Cat. No. C6198; RRID:AB_476856
Rat anti-CD31 antibody	BD Biosciences	Cat. No. 550274; RRID:AB_393571
Goat anti-VAChT antibody	Merck	Cat. No. ABN100; RRID:AB_2630394
Mouse anti-βIII-tubulin (TUJ1) antibody	Promega	Cat. No. G7121; RRID:AB_430874
Chicken anti-PGP9.5 antibody	Abcam	Cat. No. ab72910; RRID:AB_1269734
Rabbit anti-VIP antibody	Progen	Cat. No. 11428
Goat anti-GFRα2 antibody	R&D Systems	Cat. No. AF429; RRID:AB_2294621
Rabbit anti-IL6 antibody	Abcam	Cat. No. ab179570
Rabbit anti-neurturin antibody	Abcam	Cat. No. ab274417
Rat anti-RUNX2	BioLegend	Cat. No. 692802; RRID:AB_2632769
Goat anti-osteolectin	R&D Systems	Cat. No. AF3729; RRID:AB_2083418
Rabbit anti-SP7	Abcam	Cat. No. ab22552; RRID:AB_2194492
Goat anti-ALPL	Thermo Fisher Scientific	Cat. No. PA5-47419; RRID:AB_2609590
Goat anti-sclerostin	R&D Systems	Cat. No. AF1589; RRID:AB_2195345
Rabbit anti-CD51 antibody	Abcam	Cat. No. ab179475; RRID:AB_2716738
Rabbit anti-TACE antibody	Abcam	Cat. No. ab39163; RRID:AB_722563
Alexa Flour 488 donkey anti-chicken antibody	Jackson Immuno	Cat. No. 703-545-155; RRID:AB_2340375
Alexa Flour 488 donkey anti-goat antibody	Thermo Fisher Scientific	Cat. No. A11055; RRID:AB_2534102
Alexa Fluor 488 goat anti-mouse antibody	Thermo Fisher Scientific	Cat. No. A11029; RRID:AB_138404
Alexa Flour 546 donkey anti-rabbit antibody	Thermo Fisher Scientific	Cat. No. A10040; RRID:AB_2534016
Alexa Flour 647 donkey anti-rat antibody	Abcam	Cat. No. ab150155; RRID:AB_2813835
FITC donkey anti-goat antibody	Jackson Immuno	Cat. No. 705-095-003; RRID:AB_2340400
Alexa Fluor 488 donkey anti-FITC/Oregon Green antibody	Thermo Fisher Scientific	Cat. No. A11096; RRID:AB_221558
Biotin anti-mouse Ter119 antibody	BD Biosciences	Cat. No. 553672; RRID:AB_394985
Biotin anti-mouse CD45 antibody	BD Biosciences	Cat. No. 553077; RRID:AB_394607
Biotin anti-mouse CD31 antibody	BD Biosciences	Cat. No. 553371; RRID:AB_394817
Streptavidin-APC/Cy7 antibody	BD Biosciences	Cat. No. 554063; RRID:AB_10054651
CD140α (PDGFRα)-BV605 antibody	BD Biosciences	Cat. No. 740380; RRID:AB_2740111
Sca1-PE/Cy7 antibody	BioLegend	Cat. No. 122514; RRID:AB_756199
CD51-BV421 antibody	BD Biosciences	Cat. No. 740062; RRID:AB_2739827
Annexin V-FITC antibody	BioLegend	Cat. No. 640906
Alexa Fluor 647 anti-Ki67 antibody	BD Biosciences	Cat. No. 558615; RRID:AB_647130
Sclerostin antibody (in vivo inhibition)	UCB Pharma/Amgen Inc.	Scl-Ab VI, r13c7
Tocilizumab (IL-6R inhibitor)	GENENTECH	Actemra®
TACE Pro Domain (Inhibitor of ADAM17 enzyme activity)	Weizmann Institute of Science	TPD
Anti-mIL-6-IgG (soluble IL-6 inhibitor)	Invivogen	Cat. No. mabg-mil6-3
Mouse IgG Isotype control antibody	Thermo Fisher Scientific	Cat. No. 31903; RRID:AB_10959891

**Chemicals, peptides, and recombinant proteins**		

Collagen I from rat tail	Sigma-Aldrich	Cat. No. C7661
α-MEM medium	Thermo Fisher Scientific	Cat. No. 41061029
Fetal Bovine Serum	Thermo Fisher Scientific	Cat. No. 26140079
Iron-supplemented Calf Serum	Sigma-Aldrich	Cat. No. 12238C
EDTA disodium salt dihydrate	Sigma-Aldrich	Cat. No. E5134
Collagenase, type I	Sigma-Aldrich	Cat. No. C2674
β-glycerophosphate	Sigma-Aldrich	Cat. No. G5422
L-ascorbic acid phosphate	Sigma-Aldrich	Cat. No. A8960
Dexamethasone	Sigma-Aldrich	Cat. No. D4902
0.25% Collagenase, type I	Stemcell Technologies	Cat. No. 07902
Ham’s F12 medium	Thermo Fisher Scientific	Cat. No. 11765054
Horse Serum	Thermo Fisher Scientific	Cat. No. 26050070
Poly-L-ornithine	Sigma-Aldrich	Cat. No. P4957
Laminin from Engelbreth-Holm-Swarm murine sarcoma basement membrane	Sigma-Aldrich	Cat. No. L2020
Ham’s F14 medium	BioWest	Cat. No. L0138
Recombinant Human NGF	R&D systems	Cat. No. 256-GF-100
Albumax	Thermo Fisher Scientific	Cat. No. 11020-021
Progesterone	Sigma-Aldrich	Cat. No. P8783
Putrecine	Sigma-Aldrich	Cat. No. P5780
L-thyroxine	Sigma-Aldrich	Cat. No. T2501
Sodium selenite	Sigma-Aldrich	Cat. No. S5261
Triiodothyronine	Sigma-Aldrich	Cat. No. T6397
Recombinant Murine IL-6	Peprotech	Cat. No. 216-16
Recombinant Murine GDNF	Peprotech	Cat. No. 450-44
Recombinant Human Neurturin	Peprotech	Cat. No. 450-11
Recombinant Human Artemin	Peprotech	Cat. No. 450-17
Recombinant Murine Persephin	Peprotech	Cat. No. 450-35
Recombinant GFRα1 Chimera Protein (soluble GFRα1)	R&D systems	Cat. No. 560-GR
Recombinant GFRα2 Chimera Protein (soluble GFRα2)	R&D systems	Cat. No. 429-FR
DAPI	Thermo Fisher Scientific	Cat. No. D1306
TO-PRO-3	Thermo Fisher Scientific	Cat. No. T3605
RBC Lysis Buffer	BioLegend	Cat. No. 420301
Streptavidin Particles Plus	BD Biosciences	Cat. No. 557812
DMEM/F12 medium	Thermo Fisher Scientific	Cat. No. 31330
Human Endothelial SFM medium	Thermo Fisher Scientific	Cat. No. 11111-044
Chicken Embryo Extract	Methods	
N2 supplement	Thermo Fisher Scientific	Cat. No. 17502048
B27 supplement	Thermo Fisher Scientific	Cat. No. 17504-044
Recombinant Human FGF-basic	Peprotech	Cat. No. 100-18C
Recombinant Human IGF-1	Peprotech	Cat. No. 100-11
Recombinant Murine EGF	Peprotech	Cat. No. 315-09
Recombinant Human PDGF-A	Peprotech	Cat. No. 100-13A
Recombinant Human OSM	Peprotech	Cat. No. 300-10
Acetylcholine Iodide	Sigma-Aldrich	Cat. No. A7000
(-)-Nicotine	Sigma-Aldrich	Cat. No. N3876
L-Norepinephrine Hydrochloride	Sigma-Aldrich	Cat. No. 74480
Hexamethonium Bromide	Sigma-Aldrich	Cat. No. H0879
BCIP/NBT tablets (ALP detection)	Sigma-Aldrich	Cat. No. B5655
Phalloidin- Alexa Fluor 488	Bioquest	Cat. No. 23153
Triton X-100	Sigma-Aldrich	Cat. No. T8787
DAKO Fluorescence Mounting Medium	Agilent	Cat. No. S3023
IgePal 630	Sigma-Aldrich	Cat. No. I3021
BlokHen	Aves Labs	Cat. No. BH-1001
Benzyl Alcohol	Sigma-Aldrich	Cat. No. 305197
Benzyl Benzoate	Sigma-Aldrich	Cat. No. B6630
TNB (0.1 M Tris–HCl, pH7.5, 0.15 M NaCl, 0.5% blocking reagent)	Perkin Elmer	Cat. No. FP1020
Toluidine Blue	Sigma-Aldrich	Cat. No. 89640
6-Hydroxydopamine Hydrochloride	Sigma-Aldrich	Cat. No. H4381
Guanethidine Monosulfate	Sigma-Aldrich	Cat. No. BP181

**Critical commercial assays**		

SYTOX AADvanced Dead Cell Stain Kit	Thermo Fisher Scientific	Cat. No. S10349
Vectastain Elite ABC Kit	Vector Labs	Cat. No. PK-6100
Cy3-Tyramide Reagent Pack	PerkinElmer	Cat. No. SAT704A001EA
Fixation/Permeabilization Solution Kit	BD Biosciences	Cat. No. 554714
RNeasy Mini Kit	Qiagen	Cat. No. 74106
High Capacity cDNA Reverse Transcription Kit	Applied Biosystems	Cat. No. 4368814
Choline/Acetylcholine Assay Kit	Abcam	Cat. No. ab65345
TRAcP 5b ELISA kit	IDS	Cat. No. SB-TR103
DPD ELISA kit	MicroVue	Cat. No. 8007
Mouse IL-6 ELISA kit	Abcam	Cat. No. ab222503
DuoLink anti-rabbit PLUS	Merck	Cat. No. DUO92005
DuoLink anti-rabbit MINUS	Merck	Cat. No. DUO92002
DuoLink Far Red detection kit	Merck	Cat. No. DUO92013

**Experimental models: Cell lines**		

Mouse: MLO-Y4 cell line	Prof. Lynda F. Bonewald	Kato et al., 1997

**Experimental models: Organisms/strains**		

Mouse: Gfra2^-/-^	Prof. Matti S. Airaksinen	Rossi et al., 1999
Mouse: Nes-gfp	Prof. Grigori N. Eikolopov, Stony Brook, USA	Mignone et al., 2004
Mouse: CNTF^-/-^CT-1^-/-^LIF^-/-^	Prof. Michael Sendtner	Holtmann et al., 2005
Mouse: Il6^-/-^	The Jackson Laboratory	JAX: 002650
Mouse: Nrtn^-/-^	The Jackson Laboratory	JAX: 012238
Mouse: Ai14D reporter	The Jackson Laboratory	JAX: 007914
Mouse: Ai35D reporter	The Jackson Laboratory	JAX: 012735
Mouse: TH-Cre	The Jackson Laboratory	JAX: 008601
Mouse: ChAT-IRES-Cre	The Jackson Laboratory	JAX: 031661
Mouse: α7nAChRflox	The Jackson Laboratory	JAX: 026965
Mouse: LepR-Cre	The Jackson Laboratory	JAX: 008320
Mouse: C57BL/6	Charles River Laboratories	Cat# CRL027, RRID:IMSR_CRL:027
Rat: Wistar rat	Charles River Laboratories	RGD Cat# 737929,RRID:RGD_737929

**Oligonucleotides**		

Primers for mouse genotyping, see Table S1	This paper	N/A
Primers for qPCR, see Table S2	This paper	N/A

**Software and algorithms**		

GalliosTM Kaluza Software	BeckmanCoulter	RRID:SCR_016700
Incucyte Image Analysis Software	Sartorius, UK	RRID:SCR_017316
Image J/Fiji Software	National Institutes of Health	RRID:SCR_002285
Arivis Vision4D software	Arivis AG 2020	RRID:SCR_018000
Flowjo 10.6 Software	FLOWJO, LLC	RRID:SCR_008520
Analyze 14.0 Software	Analyzedirect	https://analyzedirect.com/analyze14/
GraphPad Prism 8 Software	GraphPad Software	RRID:SCR_002798

### Resource availability

#### Lead contact

Further information and requests for resources and reagents should be directed to the Lead Contact, Simon Méndez-Ferrer (sm2116@cam.ac.uk).

#### Materials availability

This study did not generate new unique reagents.

### Experimental model and subject details

#### Animals

Age and sex-matched *Gfra2*^*-/-*^ (Rossi et al., 1999), *Nes*-*gfp* (Mignone et al., 2004) (gift from G.E. Enikolopov), *CNTF*^*-/-*^*CT-1*^*-/-*^*LIF*^*-/-*^ mice (Holtmann et al., 2005), *B6.129S2-Il6*^*tm1Kopf*^*/J* (Stock No. 002650), *B6;129X1-Nrtn*^*tm1Jmi*^*/J* (Stock No. 012238), *α7nAChRflox* (Stock No. 026965), *B6.129(Cg)-Lepr*^*tm2(cre)Rck/J*^ (Stock No. 008320) (The Jackson Laboratory) and congenic CD45.2 and CD45.1 C57BL/6 mice (Charles River Laboratories) were used in this study. In some cases, *Gfra2*^*+/-*^ mice were used as controls. For genetic lineage tracing, *B6.Cg-Gt(ROSA)26Sor*^*tm14(CAG-tdTomato)Hze*^*/J* (*Ai14D*; Stock No. 007914) and *B6;129S-Gt(ROSA)26Sor*^*tm35.1(CAG-aop3/GFP)Hze*^*/J* (*Ai35D*; Stock No. 012735) reporter mice were crossed with *B6.Cg-7630403G23Rik*^*Tg(Th-cre)1Tmd*^*/J* (*TH-Cre*; Stock No. 008601) (Lindeberg et al., 2004) or *B6.129S-Chat*^*tm1(cre)Lowl*^*/MwarJ* mice (*Chat-IRES-Cre*; Stock No. 031661) (The Jackson Laboratory). Unless otherwise noted, male and female mice were distributed equally among experiments and studied at the adult stage (3-6 months). The oligonucleotide sequences used for mouse genotyping are listed in Table S1. Wistar rats (CEA, University of Seville) were used for exercise studies. Animals were housed in specific pathogen-free facilities. All animal experiments followed protocols approved by the Animal Welfare Ethical Committees, according to EU and United Kingdom Home Office regulations (PPL P0242B783).

#### Cell lines

MLO-Y4 cells (osteocyte cell line, passage 16) were seeded in 6-well plates pre-coated with collagen I (Sigma, Cat. No. C7661) and grown with α-MEM medium (ThermoFisher, Cat. No. 41061029) supplemented with 5% fetal bovine serum (FBS; ThermoFisher, Cat. No. 26140079) and 5% iron-supplemented calf serum (Sigma. Cat. No. 12238C). All cultures were maintained with 1% penicillin–streptomycin (ThermoFisher, Cat. No. 15140122) at 37 °C in a water-jacketed incubator with 5% CO_2_. Routine tests confirmed the absence of mycoplasma contamination in the cultures.

#### Osteoblast and osteocyte-like cell cultures

An illustration is provided in Figure 6A. For primary osteoblast isolation, calvaria of neonatal mice were removed on postnatal day 3 (P3) and incubated in 4mM EDTA/PBS solution for 10 minutes at 37°C with agitation. Digestion with EDTA (Sigma, Cat. No. E5134) was repeated. The supernatant was discarded and tissues were placed in 0.1% collagenase I/0.2% dispase solution (Sigma, Cat. No. C2674) for 10 minutes at 37°C with agitation. The supernatant was discarded, and enzymatic digestion was repeated four additional times (fractions II-V). Supernatant from fractions II-V were collected, washed (300xg), and cultured with αMEM supplemented with 10% FBS in 25cm^2^ flasks (1.5x10^6^cells/flask). For osteogenic differentiation to osteocyte-like cells (OLCs), passage 2 osteoblasts were grown to 90-95% confluence, followed by the addition of αMEM supplemented with 10% FBS, 5mM β-glycerophosphate (Sigma, Cat. No. G5422), 100μg/ml L-ascorbic acid phosphate (Sigma, Cat. No. A8960), and 10nM dexamethasone (Sigma, Cat. No. D4902). Osteogenic medium was changed every 3-4 days for 21 days.

#### Superior cervical ganglion (SCG) cultures

An illustration is provided in Figure 2E. SCG of neonatal mice were microdissected on postnatal day 3 (P3) and incubated with collagenase I (StemCell Technologies, Cat. No. 07902) for 20 minutes at 37°C with agitation, followed by digestion with 5% trypsin (Sigma, Cat. No. 59427C) in HBSS (ThermoFisher, Cat. No. 14175095) for 25 minutes at 37°C with agitation. Cells were washed with Ham’s F12 medium (ThermoFisher, Cat. No. 11765054) supplemented with 10% horse serum (ThermoFisher, Cat. No. 26050070), and mechanically dissociated with 200μl pipette until a single cell solution was obtained. Cells were washed (300xg), counted using hemocytometer slides, and plated on 35 mm dishes with 4 mini-wells pre-coated with poly-L-ornithine (Sigma, Cat. No. P4957) and 2% laminin (Sigma, Cat. No. L2020). Cells were plated at a density of 1x10^5^ cells per well in F14 medium (BioWest, Cat. No. L0138) supplemented with 1% penicillin/streptomycin, 40ng/ml NGF (R&D systems, Cat. No. 256-GF-100), and 2% Albumax (ThermoFisher, Cat. No. 11020-021)—a BSA solution supplemented with 60 μg/ml progesterone (Cat. No. P8783), 16 μg/ml putrecine (Cat. No. P5780), 400 ng/ml L-thyroxine (Cat. No. T2501), 38 ng/ml sodium selenite (Cat. No. S5261) and 340 ng/ml triiodothyronine (Cat. No. T6397; all from Sigma). SCG cultures were treated daily with 10ng/ml IL-6 (Peprotech, Cat. No. 216-16) with or without the addition of 10ng/ml tocilizumab (IL-6R inhibitor; Actemra) or Anti-mIL-6-IgG (sIL-6 inhibitor; Invivogen, Cat. No. mabg-mil6-3).

### Method details

#### *In vitro* growth and viability assays

MLO-Y4 cells and primary osteoblasts were plated at a density of 0.5x10^5^ cells/well on 12 well plates. Once adhered on the following day, cells were treated with 100ng/ml GDNF-family ligands, including GDNF (Cat. No. 450-44), Neurturin (Cat. No. 450-11), Artemin (Cat. No. 450-17), or Persephin (Cat. No. 450-35; all from Peprotech), and/or 300ng/ml soluble GFRα1 (R&D systems, Cat. No. 560-GR) or soluble GFRα2 (R&D systems, Cat. No. 429-FR). Cells were released after four days of treatment at approximately 90% confluency, counted using hemocytometer slides, and washed in binding buffer (0.366g/L CaCl_2_, 2.38g/L HEPES, 8.18g/L NaCl, in distilled H_2_O, pH 7.4). Once washed, cells were resuspended and stained with Annexin antibody (BioLegend, Cat. No. 640906) at 1:50 dilution for 15 minutes at room temperature. Cells were washed and stained with DAPI (ThermoFisher, Cat. No. D1306) at 1:2000 dilution, and acquired immediately using a Gallios cytometer (BeckmanCoulter). Kaluza software (BeckmanCoulter, RRID:SCR_016700) was used for analysis. Viability of day 21 osteocyte-like cells (OLCs) was measured using the SYTOX AADvanced Dead Cell Stain Kit (ThermoFisher, Cat. No. S10349), and images were acquired in live d21 cultures using the Essen Incucyte Zoom (Sartorius, UK; RRID:SCR_017316) with 20x magnification.

#### Mesensphere, CFU-OB, and CD51^+^ cell cultures

For mesensphere assays, mouse bones were crushed and digested in collagenase I at 37°C for 45 minutes with agitation. Cells were filtered through 40um mesh, spun for 5 minutes at 300xg, and resuspended in RBC lysis buffer (BioLegend, Cat. No. 420301). Negative MACS separation was performed using CD45- and Ter119-tagged magnetic nanoparticles (BD Biosciences, Cat. No. 557812) for 30-45 minutes. Following centrifugation for 5 minutes at 300xg, cells were plated on low adherence 35mm dishes (StemCell technologies, Cat. No. 27150, 1x10^6^ cells/dish) in DMEM/F12 (ThermoFisher, Cat. No. 31330)/human endothelial SFM medium (ThermoFisher, Cat. No. 11111-044) supplemented with 15% chicken embryo extract prepared as previously described (Stemple and Anderson, 1992), N2 supplement (Thermo Fisher, Cat. No. 17502048), B27 supplement (ThermoFisher, Cat. No. 17504-044), 20ng/ml hFGF, 40ng/ml hIGF-1, 20ng/ml mEGF, 20ng/ml hPDGF-A, and 20ng/ml OSM (all from Peptrotech). Spheres were passaged once, and counted after 7-10 days in culture. For CD51^+^ cell isolation and culture, BM cells were treated with collagenase I and RBC lysis buffer, and incubated with CD51-Biotin antibody (BioLegend, Cat. No. 104104; RRID:AB_313073) on ice for 30-45 minutes. Positive MACS separation was performed using CD51-tagged magnetic nanoparticles for 30-45 minutes, and 1.5-5x10^6^ cells were plated in 75cm^2^ flasks in αMEM supplemented with 20% FBS, 1% penicillin/streptomycin, 10nM dexamethasone, and 100μM ascorbic phosphate. Passage 2 CD51^+^ cells were treated daily with 10μM acetylcholine iodide (Cat. No. A7000), 10μM (-)-nicotine (Cat. No. N3876), 10μM L-norepinephrine hydrochloride (Cat. No. 74480), and/or 10μM hexamethonium bromide (Cat. No. H0879; all from Sigma). For colony-forming-unit-osteoblast (CFU-OB) assays, freshly isolated BM nucleated cells were seeded in 6-well plates (1x10^6^ cells/well) and cultured at 37 °C, 5% CO_2_ in a water-jacketed incubator in α-MEM supplemented with 15% fetal calf serum, 1% penicillin-streptomycin, and 1 mM l-ascorbic acid 2-phosphate (Sigma, Cat. No. A8960). After 28 days in culture, adherent cells were fixed with cold 4% paraformaldehyde in PBS, followed by alkaline phosphatase staining (Sigma, Cat. No. B5655). Colonies with more than 50 cells were scored as positive.

#### Immunofluorescence and confocal imaging

Bones were dissected and fixed in 4% paraformaldehyde (PFA) overnight at 4°C with shaking; for CD31 staining, fixation was performed in 2% PFA. Bones were washed and decalcified in 250mM EDTA for 7-10 days. Following decalcification, bones were washed and placed in 30% sucrose (Sigma, Cat. No. 84097) O/N at 4°C and flash-frozen in OCT compound (Fisher Scientific, Cat. No. 12-730-571). Skull bone preparation and immunofluorescence staining were performed as previously described (Ho et al., 2019). For phalloidin staining, 8-12μm-cut-tissues were rinsed with PBS, outlined using Super Pap Pen (ThermoFisher, Cat. No. 008899), blocked in 1% BSA/PBS for 1 hour in humidified chambers, and stained with phalloidin (1:500, Bioquest, Cat. No. 23153) for 48 hours at 4°C in staining solution (0.05% Triton X, 1% BSA/PBS), followed by DAPI staining for 5 minutes at room temperature with intervening washes with PBS/0.05% Triton X (Sigma, Cat. No. T8787). Coverslips were adhered using Fluorescence mounting medium (Agilent, Cat. No. S3023). Proximity ligation assays of tissue sections were performed as previously described (Kunz and Schroeder, 2019) using anti-IL6 (abcam, Cat. No. ab179570) and anti-Neurturin (abcam, Cat. No. ab274417) rabbit antibodies combined with DuoLink anti-rabbit PLUS and MINUS probes (Merck, Cat. Nos. DUO92005 and DUO92002) and Far Red detection kit (Merck, Cat. No. DUO92013). Immunofluorescent TRAP staining was performed as previously described (Jacome-Galarza et al., 2019) using ELF97 substrate (Molecular Probes E6589) with TO-PRO-3 nuclear stain (ThermoFisher, Cat. No. T3605). Nerve fiber staining was performed on half-bones according to pervious reports (Acar et al., 2015), whereby bones were longitudinally-bisected using a cryostat, blocked O/N in staining buffer (5% donkey serum, 0.5% IgePal, 10% DMSO) supplemented with 1% BlokHen (Aves Labs, Cat. No. BH-1001), and stained with primary and secondary antibodies for 3 days in staining buffer with daily intervening washes in PBS at room temperature. The following primary antibodies were used: rabbit anti-tyrosine hydroxylase (Merck, Cat. No. AB152; RRID:AB_390204), chicken anti-GFP (Aves Labs, Cat. No. GFP-1020; RRID:AB_10000240), rabbit anti-GFP (Abcam, Cat. No. ab290; RRID:AB_303395), Living Colors DsRed polyclonal antibody (which detects TdTomato, Takara/Clontech, Cat. No. 632496; RRID:AB_10013483), rat anti-Endomucin (Santa Cruz, Cat. No. sc-65495, clone V.7C7; RRID:AB_2100037), αSMA-Cy3 (Sigma, Cat. No. C6198, clone 1A4; RRID:AB_476856), rat anti-CD31 (BD Biosciences, Cat. No. 550274, Clone MEC 13.3; RRID:AB_393571), goat anti-VAChT (Merck, Cat. No. ABN100; RRID:AB_2630394), mouse anti-TUJ1 (Promega, Cat. No. G7121; RRID:AB_430874), chicken anti-PGP9.5 (Abcam, Cat. No. ab72910; RRID:AB_1269734), rabbit anti-VIP (Progen, Cat. No. 11428), goat anti-GFRα2 (R&D Systems, Cat. No. AF429; RRID:AB_2294621), rat anti-RUNX2 (BioLegend, Cat. No. 692802; RRID:AB_2632769), goat anti-osteolectin (R&D Systems, Cat. No. AF3729; RRID:AB_2083418), rabbit anti-SP7 (abcam, Cat. No. ab22552; RRID:AB_2194492), goat anti-ALPL (ThermoFisher, Cat. No. PA5-47419; RRID:AB_2609590), goat anti-sclerostin (R&D Systems, Cat. No. AF1589; RRID:AB_2195345), rabbit anti-CD51 (Abcam, Cat. No. ab179475; RRID:AB_2716738), and rabbit anti-TACE (Abcam, Cat. No. ab39163; RRID:AB_722563). The following antibodies were used for secondary and tertiary staining: Alexa Flour 488 donkey anti-chicken (Jackson Immuno, Cat. No. 703-545-155; RRID:AB_2340375), Alexa Flour 488 donkey anti-goat (ThermoFisher, Cat. No. A11055; RRID:AB_2534102), Alexa Fluor 488 goat anti-mouse (ThermoFisher, Cat. No. A11029; RRID:AB_138404), Alexa Flour 546 donkey anti-rabbit (ThermoFisher, Cat. No. A10040; RRID:AB_2534016), Alexa Flour 647 donkey anti-rat (Abcam, Cat. No. ab150155; RRID:AB_2813835), FITC donkey anti-goat (Jackson Immuno, Cat. No. 705-095-003; RRID:AB_2340400), and Alexa Fluor 488 donkey anti-FITC/Oregon Green (Thermo Fisher, Cat. No. A11096; RRID:AB_221558). VAChT staining was either amplified using FITC-Oregon Green secondary-tertiary staining or using an amplification step using Vectastain Elite ABC Kit (Vector Labs, Cat. No. PK-6100) and Cy3-Tyramide Reagent Pack (PerkinElmer, Cat. No. SAT704A001EA). Half bones were cleared after staining using 1:2 Benzyl Alcohol:Benzyl Benzoate (BABB; Sigma, Cat. Nos. 305197 and B6630), and placed on 35mm glash-bottom dishes (MatTek, Cat No. P35G-0.170-14-C) prior to sample acquisition on Zeiss LSM 710 and LSM 980 confocal microscopes with Airyscan2. Bone was imaged by second harmonic generation (2HG) with 760nm excitation, pinhole at 600μm, using a Zeiss LSM 880 multiphoton confocal microscope. For whole bone imaging, tiled z-stacks were obtained in 2μm steps up to 300μm and reconstructed using the Fiji plug-in, “Grid/Collection Stitching,” where 3-dimensional reconstruction and noise reduction were performed as previously described (Gadomski et al., 2020). For magnified images of bone and skull regions, z-stacks were obtained in 0.3-1.5μm steps up to 300μm, and maximum intensity projections from z-stacks were merged in Fiji/Image J (National Institutes of Health; RRID:SCR_002285).

#### Immunocytochemistry of SCG cultures

After 7-14 days of treatment, SCG cultures were gently fixed for 10-15 minutes in Cytofix/Cytoperm (BD, Cat. No. 554722), blocked in TNB buffer (Perkin Elmer, Cat. No. FP1020) for 1 hour at RT, stained with primary antibodies O/N at 4°C, stained with secondary antibodies for 1-2 hours at RT, followed by 5 minute DAPI stain and acquisition on Zeiss LSM 980 microscope with Airyscan2.

#### Toluidine blue staining

Following dehydration and paraffin embedding, femurs were sectioned at 7μm and rehydrated in two washes of xylenes (5min), 100% ethanol (1min), 95% ethanol (1min), and one wash in tap water (2min). Tissues were stained with 0.04% toluidine blue in acetate buffer for 4 minutes (Sigma, Cat. No. 89640), followed by two rinses in water (1min) and mounting with DPX (Sigma, Cat. No. 06522).

#### Quantitative real-time PCR (qRT-PCR)

Cells were suspended in RLT lysis buffer for RNA isolation using the RNeasy Mini Kit (Qiagen, Cat. No. 74106), and cDNA was constructed using the High Capacity cDNA Reverse Transcription Kit (Applied Biosystems, Cat. No. 4368814) per manufacturer’s instructions. No Reverse Transcriptase was added to negative controls, and samples were measured in triplicate using the Quantstudio 12K system (Applied Biosystems). Fold change and ΔΔC_T_ values were calculated in Microsoft Excel, as previously described (Schmittgen and Livak, 2008), and in some cases presented as Heatmaps using the ggplot function in RStudio. The oligonucleotide sequences used for qRT-PCR are listed in Table S2.

#### Bone marrow cell isolation and flow cytometry

Femurs and tibias were dissected, followed by the removal of muscle and tendon with a surgical scalpel, prior to isolation of central and endosteal BM fractions (see Figure S4E). For central BM fraction, a scalpel was used to cut each bone just beneath the growth plate, and the marrow cavity was flushed (25G needles, BD Biosciences) with PBS supplemented with 0.2% bovine serum albumin (Sigma, Cat. No. A4503). The removed epiphyses and flushed bones were crushed with a mortar and pestle to obtain the endosteal BM fraction. Both central and endosteal fractions were treated with 0.25% collagenase I before filtering (Stem cell technologies, Cat. No. 07902) for 30-45 minutes at 37 °C with agitation. Enzyme reaction was quenched with PBS/2% FBS solution, washed (300xg for 5 minutes), filtered using 40μm mesh, and resuspended in RBC lysis buffer (BioLegend, Cat. No. 420301), according to manufacturer’s instructions. Cells were counted using hemocytometer slides and transferred to 96-well plates for staining. The following fluorochrome-conjugated monoclonal antibodies were used for staining: Ter119-Biotin (BD Biosciences, Cat. No. 553672; RRID:AB_394985), CD45-Biotin (BD Biosciences, Cat. No. 553077; RRID:AB_394607), CD31-Biotin (BD Biosciences, Cat. No. 553371; RRID:AB_394817), Streptavidin-APCcy7 (BD Biosciences, Cat. No. 554063; RRID:AB_10054651), CD140α-BV605 (BD Biosciences, Cat. No. 740380; RRID:AB_2740111), Sca1-PEcy7 (BioLegend, Cat. No. 122514; RRID:AB_756199), CD51-BV421 (BD Biosciences, Cat. No. 740062; RRID:AB_2739827). For Ki67 staining, cells were fixed/permeabilized after surface staining in 100μl BD Cytofix/Cytoperm solution, washed twice in BD Perm/Wash buffer (BD Biosciences, Cat. No. 554714), and stained with Alexa Fluor 647 anti-Ki67 antibody (BD Biosciences, Cat. No. 558615; RRID:AB_647130). Samples were acquired using LSRFortessa (BD Biosciences) with the High Throughput Sampler (BD Biosciences) for automated sample acquisition. FACS-sorting was performed using the Influx Cell Sorter (BD Biosciences). Data was analyzed using FlowJo (Tree Star; RRID:SCR_008520) and Microsoft Excel.

#### Primary osteoblast and osteocyte isolation

Murine long bones were dissected and digested according to previous reports (Stern et al., 2012; Asada et al., 2013). Briefly, muscle and connective tissue was removed, marrow was flushed, and bones were cut into 1-2mm fragments. Bone pieces were digested in 2.5mg/ml collagenase type I (Sigma, Cat. No. C2674) for 25 minutes for the first three digestions. Osteoblasts were collected from the first two grouped fractions (Fr1+2). Cells were incubated in a 5mM EDTA solution (1% BSA/PBS) on the fourth digestion for 25 minutes. Thereafter, collagenase and EDTA digestions were alternated until 10 fractions were collected. Osteocytes were collected from the final grouped fractions (Fr7+8 or Fr9+10).

#### Bone histomorphometry

*Ex vivo* CT imaging of bones and skulls from female *Gfra2*^*-/-*^ mice (14 weeks old) was initially performed using the Skyscan model 1072 (Skyscan, Belgium) at 50kV, 191μA, 5μm voxel size. Skeletal morphometry of tibias from exercise studies (male *Gfra2*^*-/-*^ mice, 15 weeks old; female Wistar rats, 10 weeks old) was later assessed using the Mediso nanoPET/CT scanner (Mediso, Budapest, Hungary) at 80kV, 980μA, 50ms integration time, 12μm voxel size. Reconstructions were performed using the RamLak filter in Nucline software (Mediso, Budapest, Hungary). Scans were exported to Analyze 14.0 software (Analyzedirect, KS, USA) for the separation of cortical and trabecular regions. Trabecular morphometry was performed at the secondary spongiosa of the distal metaphysis. For initial studies (i.e., Figures 5A–5C), cortical morphometry was performed in the upper third segment (labelled as proximal-mid diaphysis), while later measurements (i.e., Figures S7K and S7L) were performed in the mid-diaphyseal region (Berman et al., 2019). Morphometric indices of cortical and trabecular regions were calculated using the Bone Microarchitecture Analysis Add-on, and standardized nomenclature was used for each parameter (Bouxsein et al., 2010; Dempster et al., 2013).

Dynamic histomorphometry studies in females were performed by s.c. injections of 100μl calcein (3mg/ml in NaHCO3, pH7.4) and xylenol orange (30mg/ml in NaHCO3, pH7.4) at days -10 and -2, respectively; for males, double xylenol labelling was performed at the same intervals. Nondecalcified femurs were imaged in the area 1.2-1.5 proximal to the growth plate. Non-, single-, and double-labelled trabecular surfaces, and distances between labels, were measured in ImageJ for analysis of mineral apposition rates and bone formation rates, according to established algorithms (Dempster et al., 2013).

Three-point bending tests were performed on tibias using a 4mm-distance between two holding points, and the third point directly above the tibial midshaft using Instron (model 2519-105) and BlueHill Universal analysis software for direct measurements of load and stiffness.

#### Transmission electron microscopy (TEM)

To study osteocytes, bone pieces (1-2mm^3^) were fixed in 4% paraformaldehyde; 2.5% glutaraldehyde in 0.1M sodium cacodylate (Sigma, pH 7.4) O/N at 4°C, and decalcified for 7-10 days in 250mM EDTA. Samples were post-fixed in 1% osmium tetroxide (TAAB, UK); 1.5% potassium ferricyanide (Sigma) O/N at 4°C and washed thoroughly in dH2O before staining in 3% aqueous uranyl acetate (Agar Scientific, UK) for 24h at 4°C. Tissues were dehydrated through an ethanol series, washed twice in propylene oxide (Sigma), and infiltrated with 1:1 propylene oxide:TAAB embedding resin (TAAB, UK) O/N at room temperature. Samples were subsequently immersed in fresh resin and blocks polymerised for 48h at 60°C. Thin sections of 60nm were prepared using an EM UC7 ultramicrotome (Leica Microsystems, UK) and mounted on carbon and formvar-coated 200 mesh copper grids (Agar Scientific, UK) before post-staining in 3% aqueous uranyl acetate and Reynold’s lead citrate. Samples were imaged using a Hitachi HT7800 transmission electron microscope (Hitachi High Technologies, Japan) operating at 100kV. To study nerve fibers, pre-anesthetised *Gfra2*^*+/-*^ and *Gfra2*^*-/-*^ mice were transcardially perfused with 2% formaldehyde (made from paraformaldehyde) and 2% vacuum distilled glutaraldehyde, containing 2ml/l CaCl_2_, in 0.05M sodium cacodylate buffer at 4 ⁰C and pH 7.4. Bones were then removed and postfixed by immersion in fixative solution for 24h at 4°C. Bones were decalcified for one week with several changes of 0.25 M EDTA in 20% fixative solution in dH_2_O. Specimens were sliced to 2-4mm in one dimension and fixed for an additional 4-6 hours at 4 ⁰C. Samples were rinsed for 5 x 3 minutes in cold cacodylate buffer containing 2mM CaCl_2_. They were incubated in this solution for 1% osmium ferricyanide for 18 hours, at 4 ⁰C and rinsed 5 times in DIW. This was followed by 30 minutes in 1% thiocarbohydrazide at room temperature followed by 5 more rinses in DIW. Tissues were then incubated in 1% uranyl acetate in 0.05 maleate buffer at pH 5.5 and at 4°C overnight. They were again rinsed with DIW at room temperature 5 x 3 minutes. Dehydrated through 2 times each of 50%, 70%, 90%, and 100%, ethanol. Twice in dry ethanol, 2 times in dry acetone and 2 times in dry acetonitrile. Finally, samples were infiltrated with Quetol 651 epoxy resin over a period of 5 days. The resin was cured for 48 hours at 65 ⁰C. Thin sections were prepared with a Leica Ultracut S mounted on 200 mesh copper grids and viewed with a Tecnia G2 operated at 200kV. To avoid sample loss due to overlying grid bars, blocks were mounted on SEM stubs after thin sections were taken. The blocks were coated with 20nm of carbon (Quorum instruments Q150) and the flat block faces were imaged with a concentric backscattered detector in a FEI Verios 460L ultra high resolution SEM.

#### ELISA measurements

FACS-sorted cells and digested osteoblasts/osteocytes were counted and resuspended in Choline Assay buffer for measurements of ACh content, while femurs were flushed with 1μl PBS and supernatant was collected for BM serum ACh measurements using the Choline/Acetylcholine Assay Kit, Fluorometric protocol (Abcam, Cat. No. ab65345). SCG culture supernatants were assessed using Mouse IL-6 ELISA kit (abcam, Cat. No. ab222503). ELISAs for TRAcP 5b (IDS) and DPD (MicroVue) were performed according to the manufacturers' recommendations.

#### Sympathectomy

For chemical sympathetic denervation in neonatal mice (see illustration in Figure 2A), 100 mg/kg 6-hydroxydopamine hydrochloride (6-OHDA; Sigma, Cat. No. H4381), diluted in 0.2% ascorbic acid and 0.9% NaCl, was injected subcutaneously on postnatal days P2, P4, P6, P8 and P10. For chemical sympathetic denervation in adulthood, mice were injected i.p. with 2 doses of 6-OHDA or vehicle (100 mg/kg on day 0, 250 mg/kg on day 2).

#### In vivo inhibition of IL-6

For *in vivo* IL-6 inhibition in neonatal mice (see illustration in Figure 3C), 8mg/kg tocilizumab (IL-6 receptor inhibitor) together with 0.5mg/kg anti-mIL-6-IgG (Invivogen, Cat. No. mabg-mil6-3) were injected subcutaneously once weekly for 6 weeks beginning on postnatal day P3. The same dosing protocol was employed using 8.5mg/kg mouse IgG antibody (ThermoFisher, Cat. No. 31903; RRID:AB_10959891) in control mice. Mice were sacrified one week after the final treatment for confocal analyses of nerve fibers.

#### In vivo inhibition of TACE

For in vivo TACE inhibition in neonatal mice, 2mg/kg TACE pro domain (inhibitor of ADAM17 enzyme activity) (Solomon et al., 2007) suspended in sterile PBS was injected subcutaneously 3x/week for two weeks beginning on postnatal day P3. Control mice were injected with equal volumes of PBS. Mice were sacrified at 2 weeks of age for confocal analyses of nerve fibers.

#### Hematopoietic transplantation

*LepR-Cre*;*Chrnα7*^*fl/fl*^ and control *Chrnα7*^*fl/fl*^ (CD45.2^+^) recipient mice were split dose irradiated with 12Gy, i.v. transplanted with 2 million CD45.1^+^ bone marrow nucleated cells, and were analyzed 1 month later.

#### Treadmill exercise studies and *in vivo* inhibition of sclerostin

Peripheral sympathectomy was performed on Wistar rats (University of Seville, Center for Animal Experimentation). Postnatal day 7 (P7) rats were treated subcutaneously with 50 mg/kg of guanethidine monosulfate (Sigma, Cat. No. BP181) dissolved in NaCl 0.9% (pH 7.0), 5 days per week for 3 weeks. Control rats received similar treatment with saline solution (NaCl 0.9%; pH 7.0). Two days after the last guanethidine or saline injection (P30), rats were exercised on a treadmill (TR-10, Cibertec). After two days of adaptation to the treadmill, running at 10 m/min for 15 min, rats were subjected to treadmill exercise sessions (10 m/min for 40 min, with an inclination angle of 10°) 3 days per week for 5 weeks (see illustration in Figure S7A). An electrified grid (set at 0.2 mA of intensity per pulse) was placed behind the belt of the treadmill to induce running. Sedentary rats were placed in their cages near to the treadmill in every running session. Animals were sacrificed with deep anaesthesia (120 mg/kg pentobarbital sodium; Braun) 24 hours after the last treadmill session and tissues were collected. For mouse treadmill studies, 8-12-week-old mice were subjected to 5 weeks of exercise (20 min/session, 5 days/week) on an animal treadmill (Exer 3/6 model, Columbus Instruments, USA). During the initial week, treadmill speed was set at 10m/min and progressively increased to 12.5-15m/min by the final week. Sedentary mice were placed near the treadmill during running sessions. In some mice, 8mg/kg tocilizumab (IL-6 receptor inhibitor) together with 0.5mg/kg anti-mIL-6-IgG (Invivogen, Cat. No. mabg-mil6-3) were injected subcutaneously once weekly on a rest day (see illustration in Figure 7A). Mice were sacrificed 24 hours after the final treadmill session. The same protocol was employed for sclerostin inhibition experiments in adult mice, with 25mg/kg sclerostin antibody (Scl-Ab, r13c7, UCB Pharma/Amgen Inc.) injected subcutaneously on rest days 1x/week and treadmill exercise performed 5x/week for 5 weeks.

### Quantification and statistical analysis

Area measurements of confocal/Airyscan2 images were taken from at least 3 samples using “Color Threshold” in Fiji/Image J Software to quantify positive staining and dividing by total image area. In some cases, muscle outside the periosteum was cropped from images. For phalloidin area measurements inside bone, areas outside the periosteum and endosteum were cropped for cortical bone, and areas outside trabecular surface which contained hematopoietic cells were cropped for trabecular bone. Osteocytes were quantified by manually counting DAPI^+^ phalloidin^+^ cell bodies within bone from low-magnification Airyscan2 images. Distance analyses were performed using Arivis Vision 4D software (RRID:SCR_018000) with statistical significance determined by Kolmogorov-Smirnov analysis. Data shown in figures are expressed as mean ± standard error of the mean (SEM) and are representative of at least two trials with N values representing biological replicates (animals). One Way ANOVA and Bonferroni comparison were used for multiple group comparisons, and unpaired two-tailed t tests for two-group comparisons. Significant statistical differences between groups were indicated as: ^∗^p<0.05, ^∗∗^p<0.01, ^∗∗∗^p<0.001. Statistical analyses and graphics were carried out with GraphPad Prism 8 software (RRID:SCR_002798) and Microsoft Excel.

## Data Availability

• Microscopy data reported in this paper will be shared by the lead contact upon request. • This paper does not report original code. • Any additional information required to reanalyze the data reported in this paper is available from the lead contact upon request.

## Supplemental information

Document S1. Figures S1–S7 and Tables S1 and S2

Document S2. Article plus supplemental information

## References

[bib1] Acar M., Kocherlakota K.S., Murphy M.M., Peyer J.G., Oguro H., Inra C.N., Jaiyeola C., Zhao Z., Luby-Phelps K., Morrison S.J. (2015). Deep imaging of bone marrow shows non-dividing stem cells are mainly perisinusoidal. Nature.

[bib2] Asada N., Katayama Y., Sato M., Minagawa K., Wakahashi K., Kawano H., Kawano Y., Sada A., Ikeda K., Matsui T., Tanimoto M. (2013). Matrix-embedded osteocytes regulate mobilization of hematopoietic stem/progenitor cells. Cell Stem Cell.

[bib3] Asmus S.E., Parsons S., Landis S.C. (2000). Developmental changes in the transmitter properties of sympathetic neurons that innervate the periosteum. J. Neurosci..

[bib4] Azevedo E.R., Parker J.D. (1999). Parasympathetic control of cardiac sympathetic activity: normal ventricular function versus congestive heart failure. Circulation.

[bib5] Bajayo A., Bar A., Denes A., Bachar M., Kram V., Attar-Namdar M., Zallone A., Kovács K.J., Yirmiya R., Bab I. (2012). Skeletal parasympathetic innervation communicates central IL-1 signals regulating bone mass accrual. Proc. Natl. Acad. Sci. USA.

[bib6] Beauregard C.L., Smith P.G. (1994). Parasympathetic innervation of rat peri-orbital smooth muscle: prejunctional cholinergic inhibition of sympathetic neurotransmission without direct postjunctional actions. J. Pharmacol. Exp. Ther..

[bib7] Benthem L., Mundinger T.O., Taborsky G.J. (2001). Parasympathetic inhibition of sympathetic neural activity to the pancreas. Am. J. Physiol. Endocrinol. Metab..

[bib8] Berman A.G., Hinton M.J., Wallace J.M. (2019). Treadmill running and targeted tibial loading differentially improve bone mass in mice. Bone Rep..

[bib9] Bouxsein M.L., Boyd S.K., Christiansen B.A., Guldberg R.E., Jepsen K.J., Müller R. (2010). Guidelines for assessment of bone microstructure in rodents using micro-computed tomography. J. Bone Miner. Res..

[bib10] Chowdhury S., Schulz L., Palmisano B., Singh P., Berger J.M., Yadav V.K., Mera P., Ellingsgaard H., Hidalgo J., Brüning J., Karsenty G. (2020). Muscle-derived interleukin 6 increases exercise capacity by signaling in osteoblasts. J. Clin. Invest..

[bib11] Courties A., Belle M., Senay S., Cambon-Binder A., Sautet A., Chédotal A., Berenbaum F., Sellam J. (2020). Clearing method for 3-dimensional immunofluorescence of osteoarthritic subchondral human bone reveals peripheral cholinergic nerves. Sci. Rep..

[bib12] Dempster D.W., Compston J.E., Drezner M.K., Glorieux F.H., Kanis J.A., Malluche H., Meunier P.J., Ott S.M., Recker R.R., Parfitt A.M. (2013). Standardized nomenclature, symbols, and units for bone histomorphometry: a 2012 update of the report of the ASBMR Histomorphometry Nomenclature Committee. J. Bone Miner. Res..

[bib13] Ding L., Saunders T.L., Enikolopov G., Morrison S.J. (2012). Endothelial and perivascular cells maintain haematopoietic stem cells. Nature.

[bib14] Ernsberger U., Rohrer H. (1999). Development of the cholinergic neurotransmitter phenotype in postganglionic sympathetic neurons. Cell Tissue Res..

[bib15] Espinosa-Medina I., Saha O., Boismoreau F., Chettouh Z., Rossi F., Richardson W.D., Brunet J.F. (2016). The sacral autonomic outflow is sympathetic. Science.

[bib16] Fielding C., García-García A., Korn C., Gadomski S., Fang Z., Reguera J.L., Pérez-Simón J.A., Göttgens B., Méndez-Ferrer S. (2022). Cholinergic signals preserve haematopoietic stem cell quiescence during regenerative haematopoiesis. Nat. Commun..

[bib17] Francis N.J., Asmus S.E., Landis S.C. (1997). CNTF and LIF are not required for the target-directed acquisition of cholinergic and peptidergic properties by sympathetic neurons in vivo. Dev. Biol..

[bib18] Furshpan E.J., Landis S.C., Matsumoto S.G., Potter D.D. (1986). Synaptic functions in rat sympathetic neurons in microcultures. I. Secretion of norepinephrine and acetylcholine. J. Neurosci..

[bib19] Gadomski S., Singh S.K., Singh S., Sarkar T., Klarmann K.D., Berenschot M., Seaman S., Jakubison B., Gudmundsson K.O., Lockett S., Keller J.R. (2020). Id1 and Id3 maintain steady-state hematopoiesis by promoting sinusoidal endothelial cell survival and regeneration. Cell Rep..

[bib20] García-García A., Korn C., García-Fernández M., Domingues O., Villadiego J., Martín-Pérez D., Isern J., Bejarano-García J.A., Zimmer J., Pérez-Simón J.A. (2019). Dual cholinergic signals regulate daily migration of hematopoietic stem cells and leukocytes. Blood.

[bib21] Gavioli M., Lara A., Almeida P.W., Lima A.M., Damasceno D.D., Rocha-Resende C., Ladeira M., Resende R.R., Martinelli P.M., Melo M.B. (2014). Cholinergic signaling exerts protective effects in models of sympathetic hyperactivity-induced cardiac dysfunction. PLoS One.

[bib22] Green A.C., Tjin G., Lee S.C., Chalk A.M., Straszkowski L., Kwang D., Baker E.K., Quach J.M., Kimura T., Wu J.Y. (2021). The characterization of distinct populations of murine skeletal cells that have different roles in B lymphopoiesis. Blood.

[bib23] Guidry G., Landis S.C. (1998). Target-dependent development of the vesicular acetylcholine transporter in rodent sweat gland innervation. Dev. Biol..

[bib24] Gulati G.S., Murphy M.P., Marecic O., Lopez M., Brewer R.E., Koepke L.S., Manjunath A., Ransom R.C., Salhotra A., Weissman I.L. (2018). Isolation and functional assessment of mouse skeletal stem cell lineage. Nat. Protoc..

[bib25] Habecker B.A., Landis S.C. (1994). Noradrenergic regulation of cholinergic differentiation. Science.

[bib26] Habecker B.A., Symes A.J., Stahl N., Francis N.J., Economides A., Fink J.S., Yancopoulos G.D., Landis S.C. (1997). A sweat gland-derived differentiation activity acts through known cytokine signaling pathways. J. Biol. Chem..

[bib27] Hasan W., Smith P.G. (2009). Modulation of rat parasympathetic cardiac ganglion phenotype and NGF synthesis by adrenergic nerves. Auton. Neurosci..

[bib28] Heuckeroth R.O., Enomoto H., Grider J.R., Golden J.P., Hanke J.A., Jackman A., Molliver D.C., Bardgett M.E., Snider W.D., Johnson E.M., Milbrandt J. (1999). Gene targeting reveals a critical role for neurturin in the development and maintenance of enteric, sensory, and parasympathetic neurons. Neuron.

[bib29] Hiltunen P.H., Airaksinen M.S. (2004). Sympathetic cholinergic target innervation requires GDNF family receptor GFR alpha 2. Mol. Cell. Neurosci..

[bib30] Ho Y.H., Del Toro R., Rivera-Torres J., Rak J., Korn C., García-García A., Macías D., González-Gómez C., Del Monte A., Wittner M. (2019). Remodeling of bone marrow hematopoietic stem cell niches promotes myeloid cell expansion during premature or physiological aging. Cell Stem Cell.

[bib31] Hohmann E.L., Elde R.P., Rysavy J.A., Einzig S., Gebhard R.L. (1986). Innervation of periosteum and bone by sympathetic vasoactive intestinal peptide-containing nerve fibers. Science.

[bib32] Holdsworth G., Roberts S.J., Ke H.Z. (2019). Novel actions of sclerostin on bone. J. Mol. Endocrinol..

[bib33] Holmbeck K., Bianco P., Pidoux I., Inoue S., Billinghurst R.C., Wu W., Chrysovergis K., Yamada S., Birkedal-Hansen H., Poole A.R. (2005). The metalloproteinase MT1-MMP is required for normal development and maintenance of osteocyte processes in bone. J. Cell Sci..

[bib34] Holtmann B., Wiese S., Samsam M., Grohmann K., Pennica D., Martini R., Sendtner M. (2005). Triple knock-out of CNTF, LIF, and CT-1 defines cooperative and distinct roles of these neurotrophic factors for motoneuron maintenance and function. J. Neurosci..

[bib35] Huang T., Hu J., Wang B., Nie Y., Geng J., Cheng L. (2013). Tlx3 controls cholinergic transmitter and Peptide phenotypes in a subset of prenatal sympathetic neurons. J. Neurosci..

[bib36] Ip N.Y., Nye S.H., Boulton T.G., Davis S., Taga T., Li Y., Birren S.J., Yasukawa K., Kishimoto T., Anderson D.J. (1992). CNTF and LIF act on neuronal cells via shared signaling pathways that involve the IL-6 signal transducing receptor component gp130. Cell.

[bib37] Jacome-Galarza C.E., Percin G.I., Muller J.T., Mass E., Lazarov T., Eitler J., Rauner M., Yadav V.K., Crozet L., Bohm M. (2019). Developmental origin, functional maintenance and genetic rescue of osteoclasts. Nature.

[bib38] Kato Y., Windle J.J., Koop B.A., Mundy G.R., Bonewald L.F. (1997). Establishment of an osteocyte-like cell line, MLO-Y4. J. Bone Miner. Res..

[bib82] Kunz L., Schroeder T. (2019). A 3D Tissue-wide Digital Imaging Pipeline for Quantitation of Secreted Molecules Shows Absence of CXCL12 Gradients in Bone Marrow. Cell Stem Cell.

[bib39] Landis S.C. (1976). Rat sympathetic neurons and cardiac myocytes developing in microcultures: correlation of the fine structure of endings with neurotransmitter function in single neurons. Proc. Natl. Acad. Sci. USA.

[bib40] Lazzaro L., Tonkin B.A., Poulton I.J., McGregor N.E., Ferlin W., Sims N.A. (2018). IL-6 *trans*-signalling mediates trabecular, but not cortical, bone loss after ovariectomy. Bone.

[bib41] Lindeberg J., Usoskin D., Bengtsson H., Gustafsson A., Kylberg A., Söderström S., Ebendal T. (2004). Transgenic expression of Cre recombinase from the tyrosine hydroxylase locus. Genesis.

[bib42] Loy B., Apostolova G., Dorn R., McGuire V.A., Arthur J.S., Dechant G. (2011). p38alpha and p38beta mitogen-activated protein kinases determine cholinergic transdifferentiation of sympathetic neurons. J. Neurosci..

[bib43] Luther J.A., Birren S.J. (2009). Neurotrophins and target interactions in the development and regulation of sympathetic neuron electrical and synaptic properties. Auton. Neurosci..

[bib44] Madisen L., Mao T., Koch H., Zhuo J.M., Berenyi A., Fujisawa S., Hsu Y.W., Garcia A.J., Gu X., Zanella S. (2012). A toolbox of Cre-dependent optogenetic transgenic mice for light-induced activation and silencing. Nat. Neurosci..

[bib45] Matic I., Matthews B.G., Wang X., Dyment N.A., Worthley D.L., Rowe D.W., Grcevic D., Kalajzic I. (2016). Quiescent bone lining cells are a major source of osteoblasts during adulthood. Stem Cells.

[bib46] McDonagh S.C., Lee J., Izzo A., Brubaker P.L. (2007). Role of glial cell-line derived neurotropic factor family receptor alpha2 in the actions of the glucagon-like peptides on the murine intestine. Am. J. Physiol. Gastrointest. Liver Physiol..

[bib47] McGregor N.E., Poulton I.J., Walker E.C., Pompolo S., Quinn J.M., Martin T.J., Sims N.A. (2010). Ciliary neurotrophic factor inhibits bone formation and plays a sex-specific role in bone growth and remodeling. Calcif. Tissue Int..

[bib48] Mende N., Jolly A., Percin G.I., Günther M., Rostovskaya M., Krishnan S.M., Oostendorp R.A.J., Dahl A., Anastassiadis K., Höfer T. (2019). Prospective isolation of nonhematopoietic cells of the niche and their differential molecular interactions with HSCs. Blood.

[bib49] Méndez-Ferrer S. (2019). Molecular interactome between HSCs and their niches. Blood.

[bib50] Méndez-Ferrer S., Michurina T.V., Ferraro F., Mazloom A.R., Macarthur B.D., Lira S.A., Scadden D.T., Ma'ayan A., Enikolopov G.N., Frenette P.S. (2010). Mesenchymal and haematopoietic stem cells form a unique bone marrow niche. Nature.

[bib51] Mignone J.L., Kukekov V., Chiang A.S., Steindler D., Enikolopov G. (2004). Neural stem and progenitor cells in nestin-GFP transgenic mice. J. Comp. Neurol..

[bib52] Miyashita Y., Furukawa Y., Nakajima K., Hirose M., Kurogouchi F., Chiba S. (1999). Parasympathetic inhibition of sympathetic effects on pacemaker location and rate in hearts of anesthetized dogs. J. Cardiovasc. Electrophysiol..

[bib53] Morikawa S., Mabuchi Y., Kubota Y., Nagai Y., Niibe K., Hiratsu E., Suzuki S., Miyauchi-Hara C., Nagoshi N., Sunabori T. (2009). Prospective identification, isolation, and systemic transplantation of multipotent mesenchymal stem cells in murine bone marrow. J. Exp. Med..

[bib54] Noll J.E., Williams S.A., Tong C.M., Wang H., Quach J.M., Purton L.E., Pilkington K., To L.B., Evdokiou A., Gronthos S., Zannettino A.C.W. (2014). Myeloma plasma cells alter the bone marrow microenvironment by stimulating the proliferation of mesenchymal stromal cells. Haematologica.

[bib55] Ota N., Hunt S.C., Nakajima T., Suzuki T., Hosoi T., Orimo H., Shirai Y., Emi M. (1999). Linkage of interleukin 6 locus to human osteopenia by sibling pair analysis. Hum. Genet..

[bib56] Ota N., Nakajima T., Nakazawa I., Suzuki T., Hosoi T., Orimo H., Inoue S., Shirai Y., Emi M. (2001). A nucleotide variant in the promoter region of the interleukin-6 gene associated with decreased bone mineral density. J. Hum. Genet..

[bib57] Pendry Y.D., Maclagan J. (1991). Evidence for prejunctional inhibitory muscarinic receptors on sympathetic nerves innervating guinea-pig trachealis muscle. Br. J. Pharmacol..

[bib58] Pinho S., Lacombe J., Hanoun M., Mizoguchi T., Bruns I., Kunisaki Y., Frenette P.S. (2013). PDGFRalpha and CD51 mark human Nestin+ sphere-forming mesenchymal stem cells capable of hematopoietic progenitor cell expansion. J. Exp. Med..

[bib59] Rao M.S., Landis S.C. (1990). Characterization of a target-derived neuronal cholinergic differentiation factor. Neuron.

[bib60] Robling A.G., Bonewald L.F. (2020). The osteocyte: new insights. Annu. Rev. Physiol..

[bib61] Rose-John S., Winthrop K., Calabrese L. (2017). The role of IL-6 in host defence against infections: immunobiology and clinical implications. Nat. Rev. Rheumatol..

[bib62] Rossi J., Balthasar N., Olson D., Scott M., Berglund E., Lee C.E., Choi M.J., Lauzon D., Lowell B.B., Elmquist J.K. (2011). Melanocortin-4 receptors expressed by cholinergic neurons regulate energy balance and glucose homeostasis. Cell Metab..

[bib63] Rossi J., Herzig K.H., Võikar V., Hiltunen P.H., Segerstråle M., Airaksinen M.S. (2003). Alimentary tract innervation deficits and dysfunction in mice lacking GDNF family receptor alpha2. J. Clin. Invest..

[bib64] Rossi J., Luukko K., Poteryaev D., Laurikainen A., Sun Y.F., Laakso T., Eerikäinen S., Tuominen R., Lakso M., Rauvala H. (1999). Retarded growth and deficits in the enteric and parasympathetic nervous system in mice lacking GFR alpha2, a functional neurturin receptor. Neuron.

[bib65] Saadat S., Sendtner M., Rohrer H. (1989). Ciliary neurotrophic factor induces cholinergic differentiation of rat sympathetic neurons in culture. J. Cell Biol..

[bib66] Schäfer M.K., Schütz B., Weihe E., Eiden L.E. (1997). Target-independent cholinergic differentiation in the rat sympathetic nervous system. Proc. Natl. Acad. Sci. USA.

[bib83] Schmittgen T.D., Livak K.J. (2008). Analyzing real-time PCR data by the comparative C(T) method. Nature Protocols.

[bib67] Schütz B., Schäfer M.K., Gördes M., Eiden L.E., Weihe E. (2015). Satb2-independent acquisition of the cholinergic sudomotor phenotype in rodents. Cell. Mol. Neurobiol..

[bib68] Shi Y., Oury F., Yadav V.K., Wess J., Liu X.S., Guo X.E., Murshed M., Karsenty G. (2010). Signaling through the M(3) muscarinic receptor favors bone mass accrual by decreasing sympathetic activity. Cell Metab..

[bib69] Sims N.A. (2021). Influences of the IL-6 cytokine family on bone structure and function. Cytokine.

[bib70] Smith-White M.A., Wallace D., Potter E.K. (1999). Sympathetic-parasympathetic interactions at the heart in the anaesthetised rat. J. Auton. Nerv. Syst..

[bib71] Solomon A., Akabayov B., Frenkel A., Milla M.E., Sagi I. (2007). Key feature of the catalytic cycle of TNF-alpha converting enzyme involves communication between distal protein sites and the enzyme catalytic core. Proc. Natl. Acad. Sci. USA.

[bib72] Stanke M., Duong C.V., Pape M., Geissen M., Burbach G., Deller T., Gascan H., Otto C., Parlato R., Schütz G., Rohrer H. (2006). Target-dependent specification of the neurotransmitter phenotype: cholinergic differentiation of sympathetic neurons is mediated in vivo by gp 130 signaling. Development.

[bib73] Stemple D.L., Anderson D.J. (1992). Isolation of a stem cell for neurons and glia from the mammalian neural crest. Cell.

[bib74] Stern A.R., Stern M.M., Van Dyke M.E., Jähn K., Prideaux M., Bonewald L.F. (2012). Isolation and culture of primary osteocytes from the long bones of skeletally mature and aged mice. BioTechniques.

[bib75] Taga T., Hibi M., Hirata Y., Yamasaki K., Yasukawa K., Matsuda T., Hirano T., Kishimoto T. (1989). Interleukin-6 triggers the association of its receptor with a possible signal transducer, gp130. Cell.

[bib76] Weaver C.M., Gordon C.M., Janz K.F., Kalkwarf H.J., Lappe J.M., Lewis R., O’Karma M., Wallace T.C., Zemel B.S. (2016). The National Osteoporosis Foundation’s position statement on peak bone mass development and lifestyle factors: a systematic review and implementation recommendations. Osteoporos. Int..

[bib77] Weihe E., Tao-Cheng J.H., Schäfer M.K., Erickson J.D., Eiden L.E. (1996). Visualization of the vesicular acetylcholine transporter in cholinergic nerve terminals and its targeting to a specific population of small synaptic vesicles. Proc. Natl. Acad. Sci. USA.

[bib78] Wolinsky E., Patterson P.H. (1983). Tyrosine hydroxylase activity decreases with induction of cholinergic properties in cultured sympathetic neurons. J. Neurosci..

[bib79] Yamamori T., Fukada K., Aebersold R., Korsching S., Fann M.J., Patterson P.H. (1989). The cholinergic neuronal differentiation factor from heart cells is identical to leukemia inhibitory factor. Science.

[bib80] Yang B., Slonimsky J.D., Birren S.J. (2002). A rapid switch in sympathetic neurotransmitter release properties mediated by the p75 receptor. Nat. Neurosci..

[bib81] Yue R., Shen B., Morrison S.J. (2016). Clec11a/osteolectin is an osteogenic growth factor that promotes the maintenance of the adult skeleton. eLife.

