# Supplementary material for: A cholinergic neuroskeletal interface promotes bone formation during postnatal growth and exercise
Source: Cell Stem Cell. 2022 Apr 7;29(4):528–544.e9. doi: 10.1016/j.stem.2022.02.008 (PMC9033279; doi:10.1016/j.stem.2022.02.008)
Supplement: Document S1. Figures S1–S7 and Tables S1 and S2 [file mmc1.pdf]

**Cell Stem Cell, Volume *29***

## Supplemental Information

# A cholinergic neuroskeletal interface

# promotes bone formation

# during postnatal growth and exercise

**Stephen Gadomski, Claire Fielding, Andrés García-García, Claudia Korn, Chrysa Kapeni, Sadaf Ashraf, Javier Villadiego, Raquel del Toro, Olivia Domingues, Jeremy N. Skepper, Tatiana Michel, Jacques Zimmer, Regine Sendtner, Scott Dillon, Kenneth E.S. Poole, Gill Holdsworth, Michael Sendtner, Juan J. Toledo-Aral, Cosimo De Bari, Andrew W. McCaskie, Pamela G. Robey, and Simón Méndez-Ferrer**

# Supplementary Figure S1, Related to Figure 1

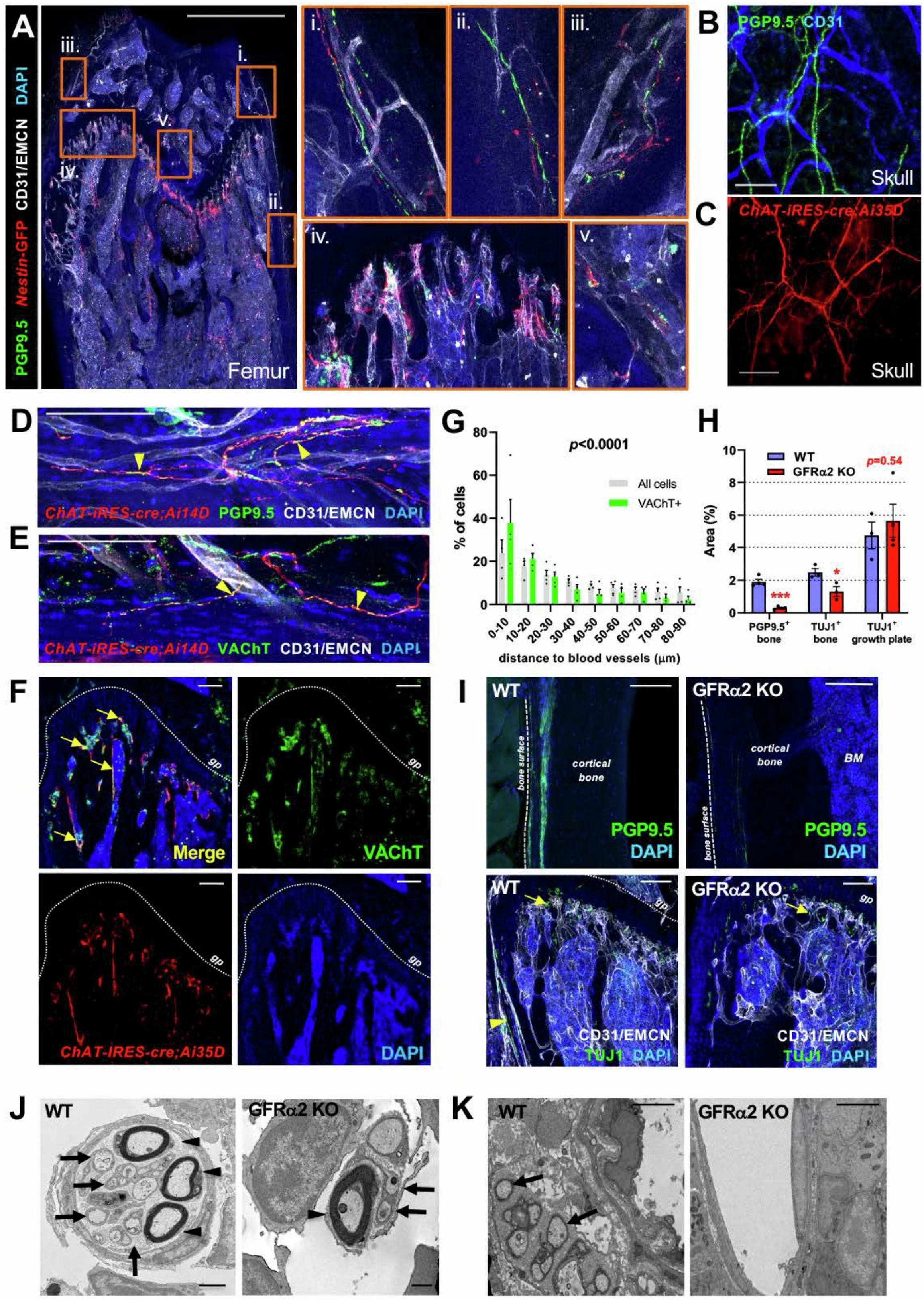


**Supplementary Figure 1. Related to Figure 1. Characterization of the cholinergic system in bone.**

(A) Immunofluorescence of *Nestin*-GFP$^+$ SSC-enriched cells (red), CD31$^+$ or Endomucin (EMCN)$^+$ blood vessels (white), and pan-neural PGP9.5 (green) in *Nes-GFP* femur. Insets show bone (i-iii) and growth plate (iv-v) areas. Scale bar, 500μm.

(B) Immunofluorescence of Protein gene product 9.5 (PGP9.5)$^+$ nerve fibers (green) and CD31$^+$ vessels (blue) in WT skull. Scale bar, 100μm.

(C) Immunofluorescence of genetically-traced cholinergic nerve fibers in *ChAT-IRES-cre;Ai35D* skull. Scale bar, 100μm. See also Fig. 1E-F.

(D) Immunofluorescence of pan-neural PGP9.5 (green), genetically-traced cholinergic fibers (red), and CD31$^+$/EMCN$^+$ blood vessels (white) in *ChAT-IRES-cre;Ai14D* periosteum. Arrowheads depict co-localization. Scale bar, 100μm.

(E-F) Immunofluorescence of vesicular acetylcholine transporter (VAChT, green), genetically-traced cholinergic cells (red), and (E) CD31$^+$/EMCN$^+$ blood vessels (white) in *ChAT-IRES-cre;Ai14D/Ai35D* bone. Neural (arrowheads) and non-neural (arrows) co-localization of VAChT and ChAT is depicted in (E) periosteum and (F) growth plate regions. Scale bars, 100μm. See also Fig. 1E-F.

(G) Distance between CD31$^+$/EMCN$^+$ blood vessels and VAChT$^+$ cholinergic fibers in cortical bone (green bars) compared to all DAPI$^+$ cells in cortical bone (grey bars). Kolmogorov-Smirnov analysis. See also Fig. 1G-H.

(H-I) Immunofluorescence (I, green) and quantification (H) of PGP9.5$^+$ or β-tubulin (TUJ1)$^+$ cells in WT or GFRα2 KO cortical bone (I, top) or proximal diaphysis (I, bottom). Scale bars, 100μm.

(J-K) Transmission electron microscopy images of control and GFRα2 KO bones. Arrows depict non- or thinly-myelinated axons, and arrowheads depict myelinated axons. Scale bars, 500 nm (J), 4μm (K).

(A, D-F, I) Nuclei were counterstained with DAPI (blue).

(G-H) Data are mean±SEM, *$p<0.05$, ***$p<0.001$, unpaired two-tailed *t* test.

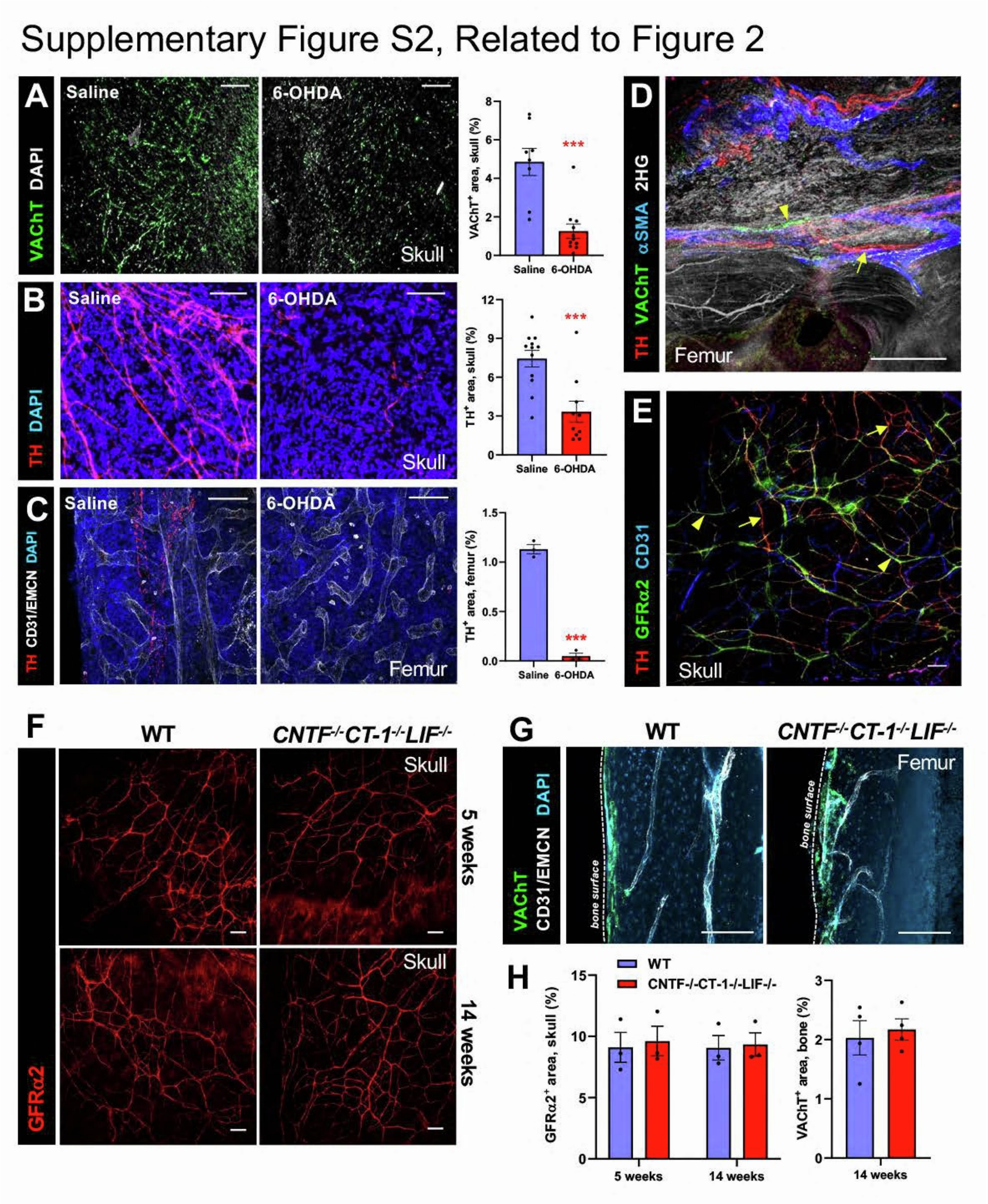


**Supplementary Figure 2. Related to Figure 2. Interleukin-6 induces a cholinergic switch in sympathetic neurons.**

(A) Immunofluorescence and quantification of vesicular acetylcholine transporter (VAChT)$^{+}$ cholinergic nerve fibers in skulls from adult mice following neonatal sympathectomy outlined in Fig. 2A. See also Fig. 2A-C.

(B-C) Immunofluorescence and quantification of tyrosine hydroxylase (TH)$^{+}$ noradrenergic nerve fibers (red) and (C) CD31$^{+}$ or endomucin (EMCN)$^{+}$ blood vessels in (B) skull and (C) femoral BM of adult mice following neonatal sympathectomy outlined in Fig. 2A. See also Fig. 2A-C.

(D) Immunofluorescence of $TH^+$ noradrenergic nerve fibers (red), $VAChT^+$ cholinergic nerve fibers (green) $\alpha$-smooth muscle actin ($\alpha$SMA)$^+$ cells (blue) and second-harmonic generation (2HG) signal of bone collagen (white) in femoral cortical bone.

(E) Immunofluorescence of $TH^+$ noradrenergic nerve fibers (red), GFR$\alpha$2$^+$ cholinergic nerve fibers (green),and vascular $CD31^+$ endothelial cells (blue) in skulls.

(D-E) Arrowheads depict cholinergic nerve fibers (green). Arrows depict noradrenergic nerve fibers (red).

(F-H) Immunofluorescence (F,G) and quantification (H) of GFRα2$^+$ or $VAChT^+$ cholinergic nerve fibers in skull bones (F) or femurs (G) from ciliary neurotrophic factor (CNTF)/cardiotrophin-1 (CT-1)/leukemia inhibitory factor (LIF) triple knockout mice.

(A-C, H) Data are mean±SEM, ***$p<0.001$.

(B-C, G) Nuclei were counterstained with DAPI.

(A-C, D-G) Scale bars,100µm.

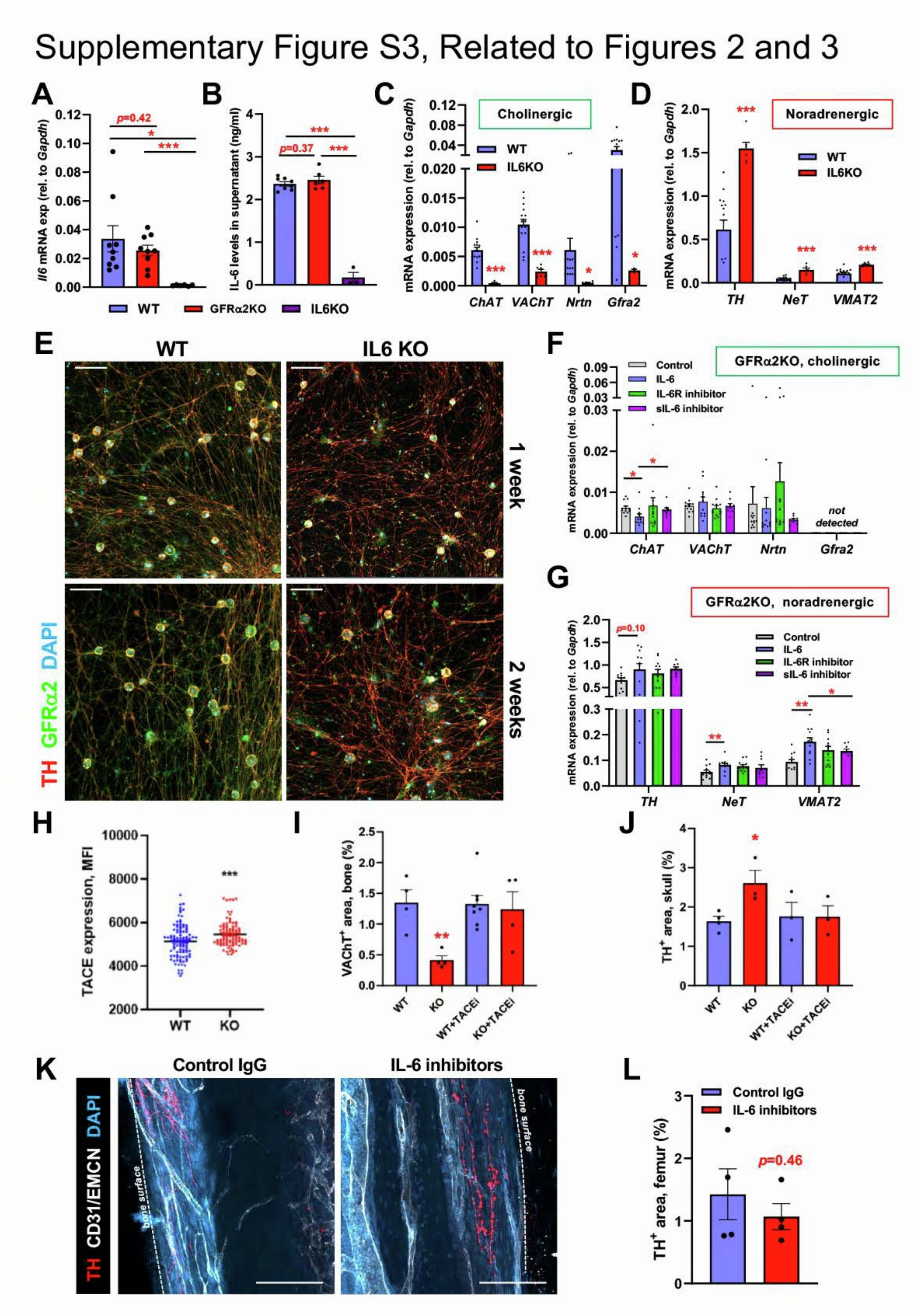


**Supplementary Figure 3. Related to Figures 2 and 3. Interleukin-6 induces a cholinergic switch of sympathetic fibers in bone.**

(A-B) qRT-PCR (A, cell extracts) and ELISA (B, culture supernatants) analyses of IL-6 expression from day 14 sympathetic superior cervical ganglion (SCG) cultures isolated from neonatal WT mice, GFRα2 KO or IL-6 KO mice. See also Fig. 2J.

(C-D) qRT-PCR analysis of (C) cholinergic and (D) noradrenergic gene expression from WT and IL6 KO SCG cultures at day 14. See also Fig. 2J.

(E) Immunofluorescence of cholinergic (GFRα2, green) and noradrenergic (tyrosine hydroxylase, TH, red) markers and nuclei counsterstaining (DAPI, blue) in SCG cultures from WT or IL-6 KO mice at days 7 and 14. Scale bars, 100μm. See also Fig. 2J.

(F-G) qRT-PCR analysis of (F) cholinergic and (G) noradrenergic gene expression from day 14 GFRα2 KO SCG cultures from treatment outlined in Fig. 2E. See also Fig. 2J.

(H) TNFα-converting Enzyme (TACE) mean fluorescence intensity (MFI) in WT or GFRα2 KO SCG cultures. Each point represents MFI of one neuron cell body.

(I-J) Bone area covered by (I) vesicular ACh transporter (VAChT)$^+$ cholinergic nerve fibers or (J) tyrosine hydroxylase (TH)$^+$ noradrenergic nerve fibers in the bones of WT or GFRα2 KO mice treated with TACE inhibitor (TACEi) or vehicle the first two postnatal weeks.

(K-L) Immunofluorescence (K) and quantification (L) of TH$^+$ noradrenergic nerve fibers (red) and CD31$^+$ or endomucin (EMCN)$^+$ blood vessels in cortical bone following IL-6 blockade outlined in Figure 3C. Scale bars, 100μm. See also Fig. 3C-F.

(A-D, F-H) Data are mean±SEM, *$p<0.05$, ***$p<0.001$. ANOVA and pairwise comparisons.

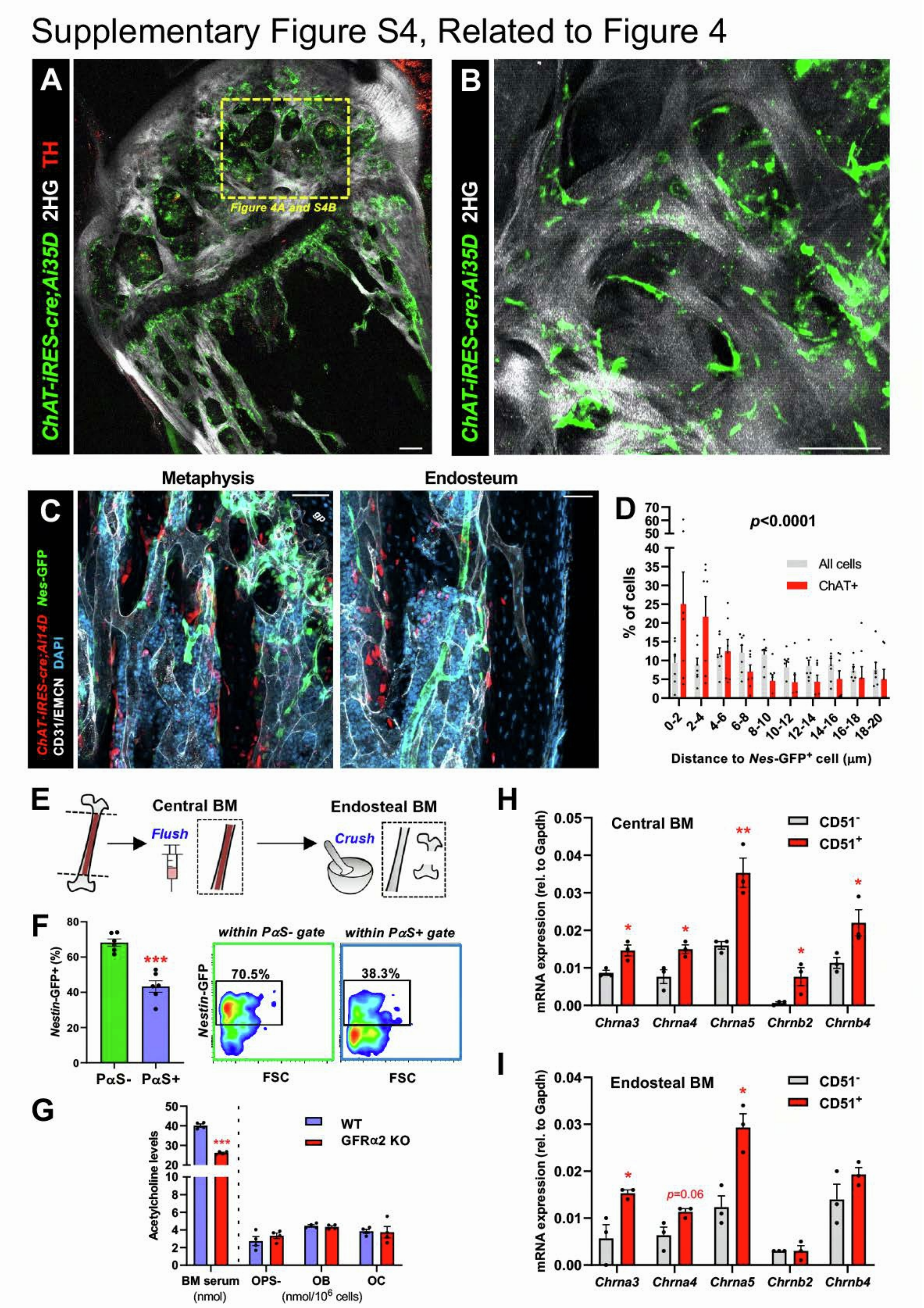


**Supplementary Figure 4. Related to Figure 4. Osteolineage cells contribute to the non-neuronal cholinergic system.**

(A-B) Low (A) and high magnification (B) images from *ChAT-IRES-cre;Ai35D* humerus, depicting ChAT$^+$ bone-lining cells (green) near the growth plate, with 2$^{nd}$ harmonic generation signal of bone collagen (2HG, white) and TH$^+$ noradrenergic nerve fibers (red) in A. Scale bars, 100µm. See also Fig. 4A-C.

(C) Genetic tracing of cholinergic bone-lining cells (red) adjacent to but distinct from *Nes*-GFP$^+$ SSC-enriched cells (green) associated with CD31$^+$/EMCN$^+$ blood vessels (white) from *ChAT-IRES-cre;Ai14D;Nes-GFP* tibias. Scale bars, 50µm. gp=growth plate. See also Fig. 4D-E.

(D) Distance between *Nestin*-GFP$^+$ SSC-enriched cells and ChAT$^+$ labelled cells in growth plate region (red bars) compared to all DAPI$^+$ cells in the same region (grey bars). Kolmogorov-Smirnov analysis. See also Fig. 4D-E.

(E) Schematic depicting isolation of central and endosteal BM fractions for flow cytometry: femurs and tibias were cut at metaphyses just beneath the growth plate and marrow was flushed for central BM fraction, while flushed bones and epiphyseal heads were crushed for endosteal BM fraction. See also Fig. 4I-J.

(F) Frequency of *Nestin*-GFP$^+$ cells among CD31$^-$CD45$^-$Ter119$^-$PDGFRα$^+$Sca1$^-$ (PαS$^-$) cells and CD31$^-$CD45$^-$Ter119$^-$PDGFRα$^+$Sca1$^+$ (PαS$^+$) cells from *Nes-GFP* mice (left) and representative flow cytometry plots (right). See also Fig. 4I-J.

(G) Acetylcholine concentration in BM supernatant (left) and in FACS-sorted CD31$^-$CD45$^-$Ter119$^-$PDGFRα$^-$CD51$^+$Sca1$^-$ (OPS$^-$) cells and digested bone fractions enriched in primary osteoblasts (OB) and osteocytes (OC) (right) from WT or GFRα2 KO mice.

(H-I) qRT-PCR analysis of nicotinic receptors in CD31$^-$CD45$^-$Ter119$^-$CD51$^+$ or CD31$^-$CD45$^-$Ter119$^-$CD51$^-$ cells isolated from central (H) and endosteal (I) BM.

(D-I) Data are mean±SEM, *p<0.05, **p<0.01, ***p<0.001, unpaired two-tailed *t* test.

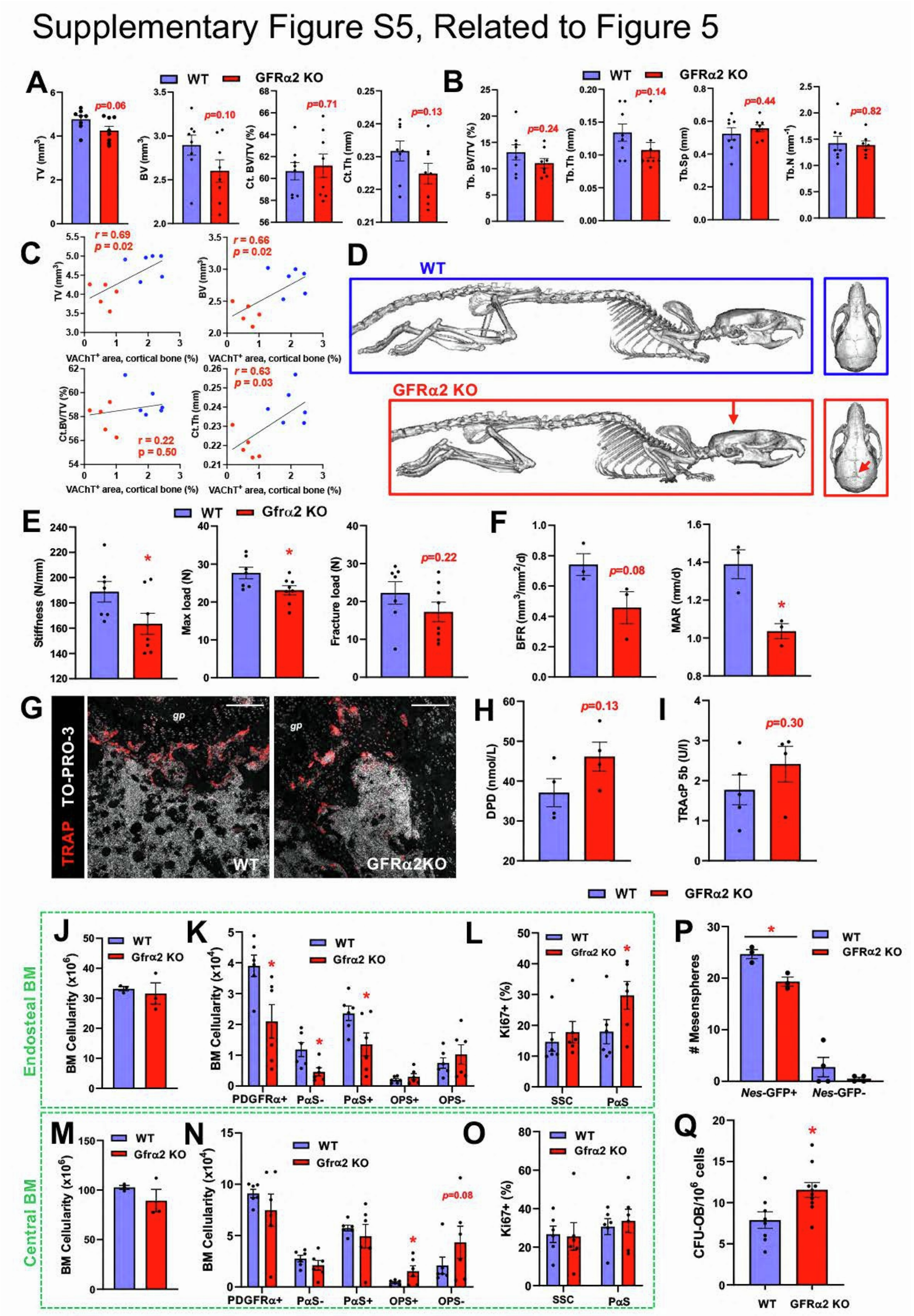


**Supplementary Figure 5. Related to Figure 5. GFRα2 loss causes reduced bone thickness and osteocyte degeneration.**

(A-B) Quantitative μCT analysis of 3D cortical (A) and trabecular (B) bone parameters in WT or GFRα2 KO male tibias: tissue volume (TV), bone volume (BV), cortical bone volume fraction (Ct.BV/TV), cortical thickness (Ct.Th), trabecular bone volume fraction (Tb.BV/TV), trabecular thickness (Tb.Th), trabecular separation (Tb.Sp), and trabecular number (Tb.N). See also Fig. 5A-B.

(C) Regression analyses of VAChT$^+$ cholinergic fiber area in cortical bone with cortical morphometry in sedentary and exercised WT (blue) and GFRα2 KO (red) mice.

(D) 3D renderings of μCT whole-body and craniofacial scans, with arrows depicting enlarged suture size and abnormal skull shape in male GFRα2 KO mice.

(E) Three-point bend analyses of tibias from WT or GFRα2 KO male mice. See also Fig. 5D.

(F) Quantification of bone formation rate (BFR) and mineral apposition rate (MAR) of femurs from WT or GFRα2 KO male mice. See also Fig. 5E-F.

(G) Immunofluorescence of TRAP$^+$ osteoclasts (red) near the growth plate from WT or GFRα2 KO BM sections. Nuclei were counterstained with TO-PRO-3 (white). Scale bars, 100μm. See also Fig. 5G.

(H-I) ELISA measurements of deoxypyridinoline cross-links (DPD, H), and the active isoform 5b of tartrate-resistant acid phosphatase (TRAcP 5b, I) from WT or GFRα2 KO BM serum.

(J-O) Flow cytometry analysis of osteolineage cells: BM cellularity (J, M), cell numbers (K, N) and proliferative (Ki67$^+$) fraction (L, O) of CD31$^-$CD45$^-$Ter119$^-$PDGFRα$^+$Sca1$^-$ (PαS$^-$), CD31$^-$CD45$^-$Ter119$^-$PDGFRα$^+$Sca1$^+$ (PαS$^+$) cells, CD31$^-$CD45$^-$Ter119$^-$PDGFRα$^-$CD51$^+$Sca1$^+$ (OPS$^-$), and CD31$^-$CD45$^-$Ter119$^-$PDGFRα$^-$CD51$^+$Sca1$^-$ (OPS$^+$) cells within endosteal (J-L) and central (M-O) BM fractions.

(P) Primitive self-renewing mesenchymal spheres (mesenspheres) from *Nes*-GFP$^{+/-}$ cells from GFRα2-competent or KO mice.

(Q) Colony-forming-units-osteoblast (CFU-OB) from WT or GFRα2 KO mice.

(A-B, E-F, H-Q) Data are mean±SEM, *p<0.05, unpaired two-tailed *t* test.

# Supplementary Figure S6, Related to Figures 5 and 6

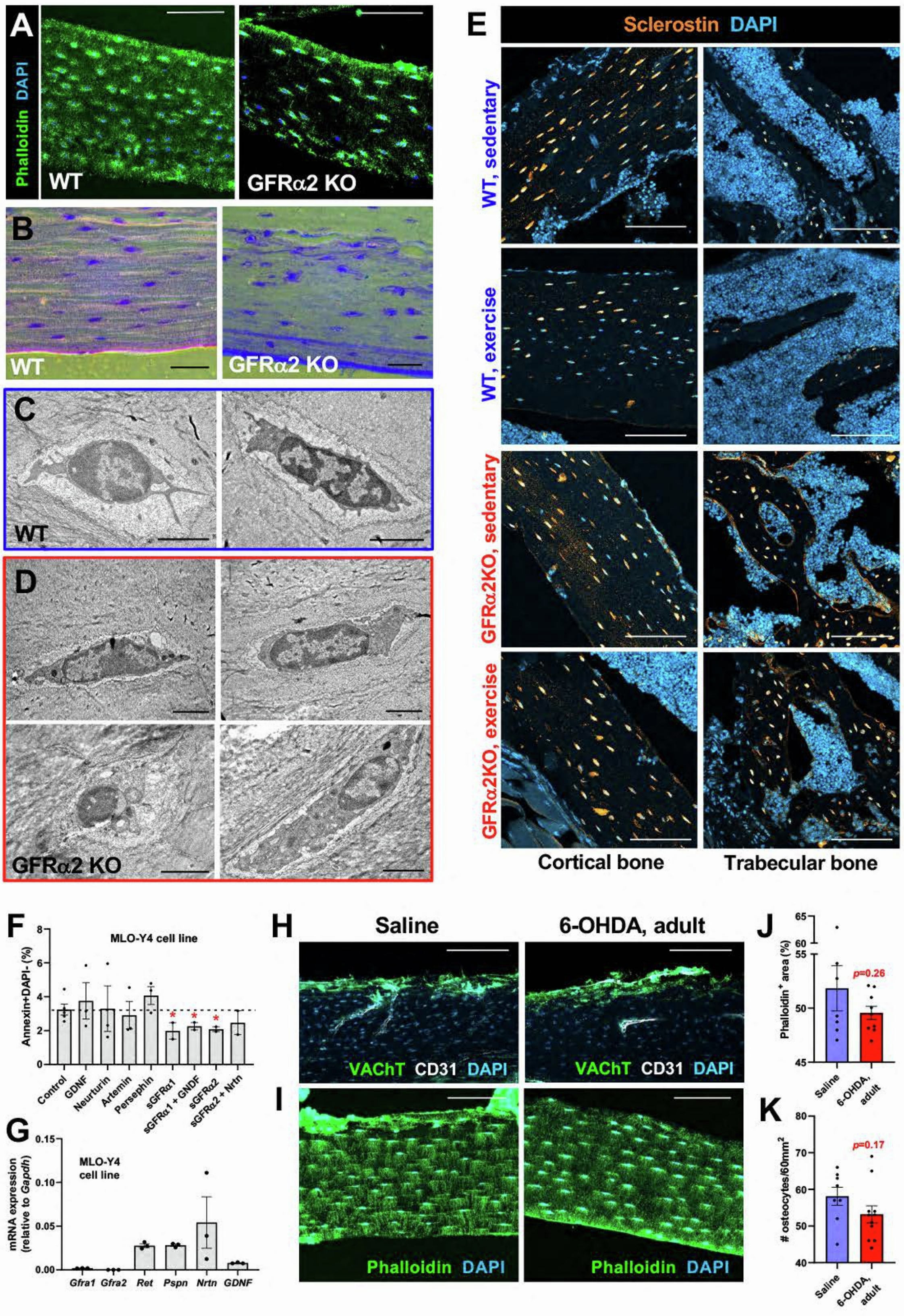


**Supplementary Figure 6. Related to Figure 6. GFRα2 signaling maintains osteocyte connectivity and survival.**

(A) Phalloidin-stained (green) osteocytes in femurs from WT or GFRα2 KO mice. Nuclei were counterstained with DAPI (blue). Scale bars, 100μm. See also Fig. 6C.

(B) Toluidine blue stains of cortical bone from WT or GFRα2 KO femurs. Scale bars, 50μm.

(C-D) Transmission electron micrographs of osteocytes from WT (C) or GFRα2 KO (D) humeri. Scale bars, 2μm. See also Fig. 5L.

(E) Immunofluorescence of sclerostin (orange) in osteocytes and DAPI-stained nuclei (blue) in femoral BM sections from WT or GFRα2 KO mice subjected to treadmill exercise for 5 days/wk for 5 wks. Scale bars, 100μm.

(F) Frequency of apoptotic MLO-Y4 osteocyte-like cells after 4 days of treatment with GDNF-family ligands and soluble receptors.

(G) qRT-PCR analysis of GDNF-family ligands and receptors in MLO-Y4 cells.

(H) Immunofluorescence of $VAChT^+$ cholinergic nerve fibers (green), $CD31^+$ blood vessels (white) and DAPI-stained nuclei (blue) in femoral cortical bone from adult WT mice subjected to chemical sympathectomy (6-OHDA) during adulthood. Scale bars, 100μm.

(I-K) Fluorescence (I, green) and quantification of $phalloidin^+$ (J) osteocytes (K) from adult WT mice subjected to chemical sympathectomy (6-OHDA) during adulthood. Scale bars, 100μm. See also Fig. 6L-N.

(F-G, J-K) Data are mean±SEM, *$p<0.05$, unpaired two-tailed *t* test.

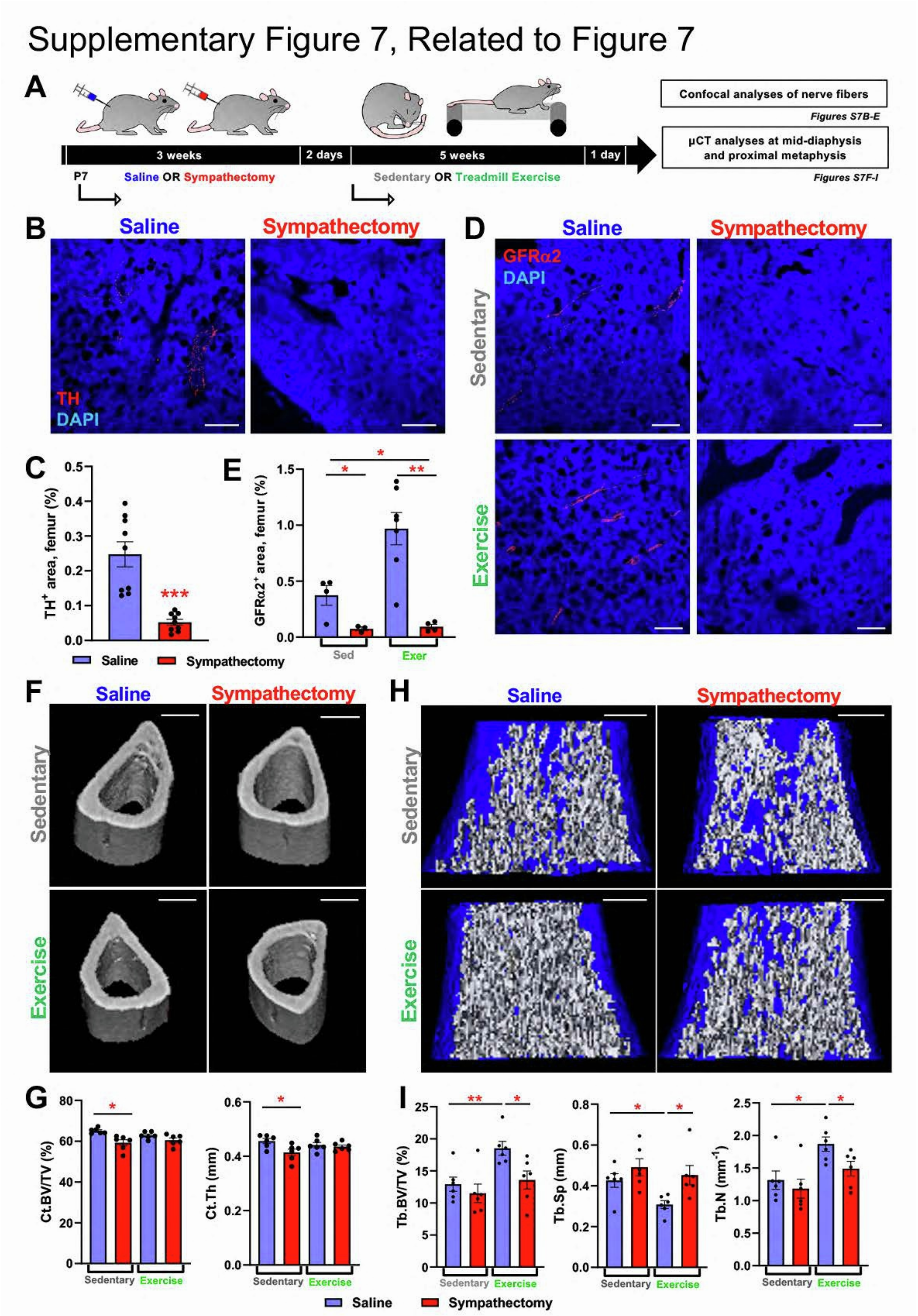


**Supplementary Figure 7. Related to Figure 7. Moderate exercise increases bone cholinergic innervation through sympathetic cholinergic fibers.**

(A) Moderate exercise schematic: Wistar rats were treated with guanethidine monosulfate (sympathectomy) or saline from postnatal day 7 (P7) for 3 weeks (5d/week), followed by treadmill exercise for 5 weeks (3d/week).

(B-C) Immunofluorescence (B, red) and quantification (C) of tyrosine hydroxylase $(TH)^{+}$ noradrenergic nerve fibers in BM sections of control or sympathectomized sedentary rats. Scale bars,100μm.

(D-E) Immunofluorescence (D, red) and quantification (E) of $GFR\alpha2^{+}$ cholinergic nerve fibers in sedentary/exercised control/sympathectomized rats. Scale bars,100μm.

(B, D) Nuclei were counterstained with DAPI (blue).

(F) 3D renderings from μCT scans of tibial mid-diaphyseal region in sedentary and exercised rats. Scale bars, 1mm.

(G) Quantitative μCT analysis of cortical bone volume fraction (Ct.BV/TV) and thickness (Ct.Th) from sedentary/exercised control/sympathectomized female rats.

(H) 3D renderings of μCT scans of tibial upper-diaphyseal region just beneath growth plate depicting cortical bone (blue) and trabecular bone (white). Scale bars, 1mm.

(I) Quantitative μCT analysis of trabecular bone volume fraction (Tb. BV/TV), thickness (Tb.Th), and separation (Tb.Sp) from sedentary/exercised control/sympathectomized female rats.

(C, E, G, I) Data are mean±SEM, $*p<0.05$, $**p<0.01$, $***p<0.001$; unpaired two-tailed *t* test (C) and ANOVA and pairwise comparisons (E, G, I).

**Table S1.**
Oligonucleotide sequences used for mouse genotyping.

| Gene | Primer Sequence (5'-3') |
|---|---|
| *Gfra2 P1* | CACATACACACAAAACTGTGGG |
| *Gfra2 P2* | ATTCGCAGCGCATCGCCTTC |
| *Gfra2 P3* | ATGTTGGAAGTCTCCTTCTC |
| *GFP P1* | ATCATGGCCGACAAGCAGAAGAAC |
| *GFP P2* | GTACAGCTCGTCCATGCCGAGAGT |
| *Cre P1* | AATGCTTCTGTCCGTTTGCCGGT |
| *Cre P2* | CCAGGCTAAGTGCCTTCTCTACA |
| *tdTomato P1* | AGCAAGGGCGAGGAGGTCATC |
| *tdTomato P2* | CCTTGGAGCCGTACATGAACTGG |
| *IL6 P1* | TTCCATCCAGTTGCCTTCTTGG |
| *IL6 P2* | TTCTCATTTCCACGATTTCCCAG |
| *IL6 P3* | CCGGAGAACCTGCGTGCAATCC |

**Table S2.**
Oligonucleotide sequences used for quantitative real-time RT-PCR.

| Gene | Forward (5'-3') | Reverse (5'-3') |
|---|---|---|
| *ChAT* | GCCAGTGGAAGAATCGTCAT | TTGTGCATGTGAGTGTGTGG |
| *Cx43* | CCCGAACTCTCCTTTTCCTT | TGGGCACCTCTCTTTCACTT |
| *Dmp1* | GGTTTTGACCTTGTGGGAAA | TTGGGATGCGATTCCTCTAC |
| *E11* | CTAACCACCACTCCCACTT | CCAATAGACTCCAACCTGAAGA |
| *Gapdh* | CAGCAAGGACACTGAGCAA | TATTATGGGGGTCTGGATG |
| *GDNF* | GCCCTTCGCGCTGAGCAGTGAC | GTCGTACGTTGTCTCAGCTGC |
| *Gfra1* | TCCAATGTGTCGGGCAATAC | GGAGGAGCAGCCATTGATTT |
| *Gfra2* | TTTAACATGATCTTGGCAAACG | AGCGGAGGGTTTCGTCTAA |
| *Gfra3* | GTGTGAAATGCTGGAAGGGT | TCAGGAGCAGAATCAAGGGA |
| *Gfra4* | CTCTCCATACTTCCTGTCCT | CTACAAAAGTGACCCTCTCC |
| *Il6* | TAGTCCTTCCTACCCCAATTTCC | TTGGTCCTTAGCCACTCCTTC |
| *Mepe* | GATGCAGGCTGTGTCTGTTG | TGTCTTCATTCGGCATTGG |
| *MT1MMP* | CCCTAGGCCTGGAACATTCT | TTTGGGCTTATCTGGGACAG |
| *NeT* | AACTTCAAGCCGCTCACCTA | ATGACATAGGCAGGGACCAG |
| *Nrtn* | CAGCGGAGGCGCGTGCGCAGAGAGCG | TAGCGGCTGTGCACGTCCAGGAAGGACACCT |
| *Phex* | TGCCAGAGAACAAGTGCAAA | CTAATGGCACCATTGACCCTA |
| *Pspn* | TGAGAGCAGCAAGAGTACAAACTCA | CTCGCACTCAGGAGGCTGTAG |
| *Ret* | GCTGCATGAGAATGACTGGA | TGGCATTCTCCCTCTCTCTG |
| *Sost* | AGCCTTCAGGAATGATGCCAC | CTTTGGCGTCATAGGGATGGT |
| *TH* | GTGCCAGAGAGGACAAGGTTC | CGATACGCCTGGTCAGAGA |
| *VAChT* | TCACTCACTTGGCTTTGAGC | GGTTCATCAAGCAGCACATC |
| *VMAT2* | GCGAGCATCTCTTATCTCATTGG | AAATGCTGATCCCAACAACTATCA |
